# Prediction of Contact Residue Pairs Based on Co-Substitution between Sites in Protein Structures

**DOI:** 10.1371/journal.pone.0054252

**Published:** 2013-01-16

**Authors:** Sanzo Miyazawa

**Affiliations:** Graduate School of Engineering, Gunma University, Kiryu, Gunma, Japan; University of Lausanne, Switzerland

## Abstract

Residue-residue interactions that fold a protein into a unique three-dimensional structure and make it play a specific function impose structural and functional constraints in varying degrees on each residue site. Selective constraints on residue sites are recorded in amino acid orders in homologous sequences and also in the evolutionary trace of amino acid substitutions. A challenge is to extract direct dependences between residue sites by removing phylogenetic correlations and indirect dependences through other residues within a protein or even through other molecules. Rapid growth of protein families with unknown folds requires an accurate *de novo* prediction method for protein structure. Recent attempts of disentangling direct from indirect dependences of amino acid types between residue positions in multiple sequence alignments have revealed that inferred residue-residue proximities can be sufficient information to predict a protein fold without the use of known three-dimensional structures. Here, we propose an alternative method of inferring coevolving site pairs from concurrent and compensatory substitutions between sites in each branch of a phylogenetic tree. Substitution probability and physico-chemical changes (volume, charge, hydrogen-bonding capability, and others) accompanied by substitutions at each site in each branch of a phylogenetic tree are estimated with the likelihood of each substitution, and their direct correlations between sites are used to detect concurrent and compensatory substitutions. In order to extract direct dependences between sites, partial correlation coefficients of the characteristic changes along branches between sites, in which linear multiple dependences on feature vectors at other sites are removed, are calculated and used to rank coevolving site pairs. Accuracy of contact prediction based on the present coevolution score is comparable to that achieved by a maximum entropy model of protein sequences for 15 protein families taken from the Pfam release 26.0. Besides, this excellent accuracy indicates that compensatory substitutions are significant in protein evolution.

## Introduction

The evolutionary history of protein sequences is a valuable source of information in many fields of science not only in evolutionary biology but even to understand protein structures. Residue-residue interactions that fold a protein into a unique three-dimensional (3D) structure and make it play a specific function impose structural and functional constraints in varying degrees on each amino acid. Selective constraints on amino acids are recorded in amino acid orders in homologous protein sequences and also in the evolutionary trace of amino acid substitutions. Negative effects caused by mutations at one site must be compensated by successive mutations at other sites [1]–[4], otherwise negative mutants will be eliminated from a gene pool and never reach fixation in a population, causing coevolution between sites [5]–[8]. Such structural and functional constraints arise from interactions between sites mostly in close spatial proximity. Thus, it is suggested and also has been shown that the types of amino acids [9]–[16] and amino acid substitutions [6]–[8], [17]–[31] are correlated between sites that are close in a protein 3D structure. Since protein families with unknown folds are growing as genome and metagenome projects proceed with next-generation sequencing technologies, it is needed to not only show the fact of coevolution between closely-located sites in protein structure but also to accurately predict contact residue pairs enough to achieve reasonable protein structure prediction [15], [16], [32]. However, correlations of amino acid types and amino acid substitutions result from not only direct but also indirect dependences through other residues within a protein or even through other molecules involved in a molecular complex [33], [34] such as oligomerization [28], protein-substrate, protein-protein [12], and protein-DNA. Also, phylogenetic correlation must be taken into account, especially in the correlation analysis of amino acid type in a multiple sequence alignment; otherwise false indications of coevolution may be led [20], [30]. In addition, statistical noise originating from a small number of homologs and methodological limitations are obstacles to decode correlations into spatial relationships between sites. However, protein families consisting of homologous sequences in a wide range of divergence are now collected in protein family databases such as Pfam [35], and become available to reduce statistical noise to a sufficiently small amount. A present challenge is thus to extract only direct dependences between sites by excluding indirect correlations between them from a wide variety of homologous sequences evolutionarily exploited in a sequence space [11], [14]–[16], [29], [32].

Extracting essential information from the evolutionary sequence record have been attempted using global statistical models. A Bayesian graphical model was applied to disentangling direct from indirect dependencies between residue positions in multiple sequence alignments of proteins [11], and a significant improvement was achieved in the accuracy of contact prediction [14]. A Bayesian graphical model was also applied to the analysis of the joint distribution of substitution events to identify significant associations among residue sites [29]. Recently, remarkable accuracy of contact prediction was achieved [15], [16] by using a maximum entropy model [12] of a protein sequence, constrained by the statistics of a multiple sequence alignment, to infer residue pair coupling. Partial correlation coefficients derived from mutual information of residue pair coupling were also used to extract direct information [32]. They developed not only a robust method to extract essential correlations of amino acid type between residue positions in multiple sequence alignments, but also showed that inferred residue-residue proximities can be sufficient information to predict a protein fold without the use of known three-dimensional structures.

Here, we report an alternative approach of inferring coevolving site pairs from concurrent and compensatory substitutions between sites in each branch of a phylogenetic tree. First, for each protein family, its phylogenetic tree 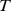 calculated by the FastTree [36] is taken from the Pfam database [35] and branch lengths 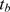 of the tree are optimized by maximizing the likelihood of the tree in a mechanistic codon substitution model [37], [38]. in a mechanistic codon substitution model [37], [38]. The variation of selective constraints over sites is approximated by a discrete gamma distribution [39]. Then, substitution probability and mean changes of physico-chemical properties of side chain accompanied by amino acid substitutions at each site in each branch of the tree are estimated with the likelihood of each substitution to detect concurrent and compensatory substitutions. Dutheil et al. [7] named such quantities along branches substitution vectors and used Pearson's correlation coefficients between substitution vectors to detect coevolving positions in a molecule. Here, instead of Pearson's correlation coefficients that reflect not only direct but also indirect dependences between sites, partial correlation coefficients of their characteristic changes accompanied by substitutions along branches between sites are employed to remove a linear multiple dependence on characteristic changes along branches at other sites. In other words, a Gaussian graphical model [40] is assumed for site dependence, because a conditional independence between two variables given other variables in a multi-variate Gaussian distribution is equivalent to zero partial correlation coefficient between the two variables. It is demonstrated that unlike Pearson's correlation coefficients partial correlation coefficients well reflect direct dependences between sites, indicating that improper correlations such as indirect and phylogenetic correlations included in Pearson's correlation coefficients are well removed in the partial correlation coefficients. Then, coevolution scores are defined on the basis of partial correlation coefficients of the various types of characteristic quantities. It was pointed out that considering compensatory substitutions [8] and substitutions affecting physico-chemical properties [5] are useful for detecting coevolving site pairs. Here, in addition to substitution probability that is a primary quantity, we consider various kinds of physico-chemical changes of amino acid accompanied by a substitution, which are not only volume, charge, and hydrophobicity as used in [8], but also hydrogen-bonding capability, 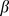 and turn propensities, the capability of aromatic interaction, branched side-chain, and cross-link capability. It is shown that compensatory substitutions can be well detected by finding the negative direct correlation of side-chain volume, charge, or hydrogen-bonding capability in concurrent substitutions. The direct correlations of other physico-chemical properties listed above are also shown to be useful to detect coevolution between sites. Then, coevolving site pairs are inferred in the decreasing order of the overall coevolution score. Accuracy of contact prediction based on the overall coevolution score is comparable to that by a maximum entropy model [16] of protein sequences, which was shown to be more accurate than other prediction methods (mutual information, statistical coupling analysis [9], [13], and Bayesian network model [14] ), for 15 protein families of the four major SCOP fold classes taken from the Pfam release 26.0 [35], indicating that the present method can be an alternative approach for contact prediction. Also, a fact that contact site pairs can be well predicted by the present method strongly indicates that compensatory substitutions are significant in protein evolution, because the present method based on concurrent and compensatory substitutions will not work at all if all substitutions are completely neutral.

## Methods

### Mean of Characteristic Changes Accompanied by Substitutions at Each Site in Each Branch of a Phylogenetic Tree in a Maximum Likelihood Model

Assuming that substitutions occur independently at each site, a likelihood 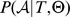 of a sequence alignment 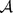 in a phylogenetic tree 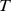 under a evolutionary model 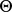 is represented as a product over sites of the likelihood of a sequence alignment 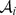 for site 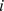.
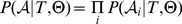
(1)

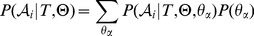
(2)where the *a priori* probability distribution of a parameter 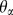 for the variation of selective constraint [37], [38] is assumed to be equal to 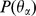, Here, a mechanistic codon substitution model [37], [38] is used as the evolutionary model 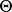. Then, if substitutions are assumed to be in the equilibrium state of a time-reversible Markov process, the likelihood of a sequence alignment 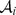 for site 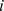 will be calculated by taking any node as a root node. Let us assume here that the root node is a left node (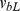) of a branch 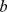.



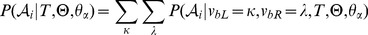
(3)

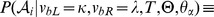


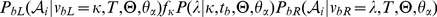
(4)where 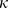 and 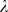 depending on the evolutionary model correspond to the type of codon in the present codon substitution model, and 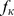 is the equilibrium frequency of 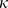. 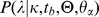 is a substitution probability from  to 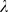 at the branch 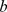 whose length is equal to 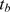. 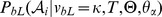 is a conditional likelihood of the left subtree with 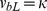 [41]. In the maximum likelihood (ML) method for phylogenetic trees, the tree 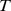 and parameters 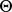 are estimated by maximizing the likelihood.



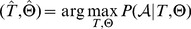
(5)Then, the mean 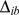 of any quantity 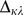 accompanied by substitutions from 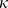 to 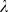 at each site 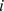 in each branch 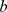 can be calculated as follows; 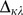 corresponds to characteristic changes for coevolution such as volume and charge changes due to amino acid substitutions, and is defined by Eqs. 12–22 in the next section.
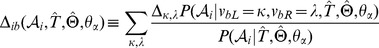
(6)

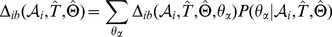
(7)where 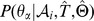 is a posterior probability calculated from



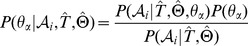
(8)A Bayesian method for mapping mutations on a phylogenetic tree was first discussed by Nielsen [42], and the present formulation of Eqs. 6 and 7 was introduced as a substitution vector along branches at site 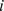 by Dutheil et al. [7] for detecting coevolving positions in a molecule. The method named substitution mapping for mapping evolutionary trajectories of discrete traits on phylogenies was further extended [43]–[45], and was shown to provide extremely robust statistics [46], [47].

### Pearson's and Partial Correlation Coefficients of Feature Vectors between Sites

If 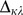 is defined to be equal to 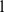 for 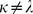 and 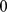 for 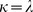, 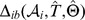 will represent the expected value of substitution probability at site 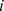 in branch 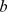. Let us define a vector 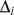 as follows, and consider the correlation of the two vectors, 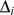 and 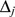.
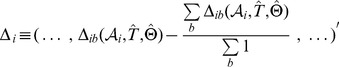
(9)where 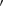 denotes the transpose of a matrix. A correlation matrix 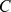 is defined to be a matrix whose 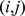 element is the correlation coefficient 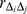 between 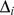 and 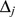.
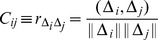
(10)where 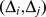 denotes the inner product of the two vectors.

The correlation between sites 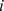 and 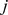 may be an indirect correlation resulting from correlations between sites 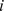 and 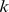 and between sites 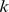 and 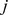. To remove such indirect correlations, partial correlation coefficients are used here. The partial correlation coefficient is a correlation coefficient between residual vectors (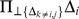 and 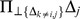) of given two vectors that are perpendicular to a subspace consisting of other vectors except those two vectors (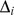 and 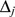) and therefore cannot be accounted for by a linear multiple regression on other vectors; 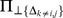 is a projection operator to a space perpendicular to the subspace. If the correlation matrix is regular, then the partial correlation coefficients 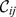 will be related to the 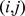 element of its inverse matrix.
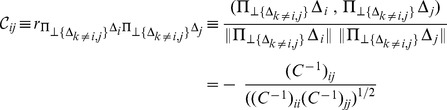
(11)


### Characteristic Variables Indicating Coevolution between Sites

The following characteristic changes accompanied by substitutions whose correlations indicate coevolution between sites have been used as 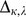 in Eq. 6.

1. Occurrence of amino acid substitution.

The most primary quantity is one (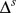) that is defined as follows and indicates the substitution probability of amino acid at a site.
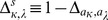
(12)where 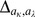 is the Kronecker's 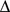 that takes 1 if 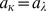 and 0 otherwise. The 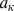 is the type of amino acid corresponding to 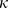, which denotes the type of codon in the present codon model. 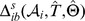 in Eq. 7 indicates the expected value of the probability of amino acid substitution at site 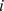 in branch 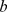. This quantity was also used [7], [19], [29] for the prediction of contact residue pairs in protein structures.

2. Change of a side chain volume accompanied by an amino acid substitution.

Protein structures must be tightly packed [48], and therefore mutations between amino acids whose side chain volumes significantly differ from each other tend to unstabilize protein structures and therefore will be eliminated from a gene pool by selection [49] unless the volume change is compensated by successive mutations at sites closely located in protein structures. Thus, the volume changes of side chains caused by amino acid substitutions are used to detect coevolution between closely located sites in protein structures.
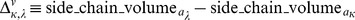
(13)where 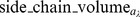 means the volume of side chain 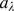. The amino acid volumes used here are the mean volume occupied by each type of amino acid in protein structures, and taken from the set named BL+ in the Table 6 of [50]; the volume of a half cystine (labeled as “cys” in the table) is used here for a cysteine.

3. Change of a side chain charge accompanied by an amino acid substitution.

Charge-charge interactions in protein structures are known to be significant. Substitutions that keep favorable charge-charge interactions are expected to be advantageous in selection.
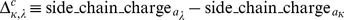
(14)where 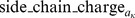 represents a charge of side chain type 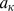 and takes 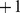 for positively charged side chains (arg and lys), 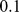 for his, and 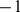 for negatively charged ones (asp, glu).

4. Change of hydrogen-bonding capability accompanied by an amino acid substitution.

One of the most important interactions to stabilize protein structures is a hydrogen-bonding interaction. Substitutions that keep hydrogen-bonds will be advantageous in selection. In order to detect whether hydrogen-bonds between side chains can be kept despite substitutions, the change of hydrogen-bonding capability is defined here as.
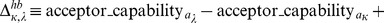


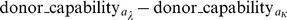
(15)where 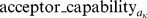 takes 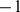 if a side chain 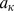 can be an hydrogen-bonding acceptor and 0 otherwise. 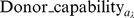 takes 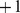 if a side chain 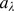 can be a hydrogen-bonding donor and 0 otherwise. Hydrogen-bonding acceptors are asn, asp, gln, glu, his, ser, thr, and tyr. Hydrogen-bonding donors are arg, asn, gln, his, lys, ser, thr, trp, and tyr. A negative correlation is expected for this quantity between closely located sites in a protein 3D structure.

5. Change of hydrophobicity accompanied by an amino acid substitution.

Also, hydrophobic interactions are crucial for a polypeptide chain to be folded into a unique three-dimensional structure. Hydrophobic interactions may be correlated between substitutions at nearby sites in a protein 3D structure.
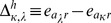
(16)where 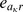 is the mean contact energy of an amino acid 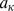 with surrounding residues (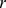) in protein structures; see [51] for its exact definition.

6. Change of 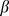 propensities accompanied by an amino acid substitution.

Changes of 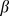 and turn propensities [52] are also examined. The change of 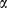 propensity [52] (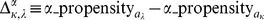) is also examined but it is not used to define the overall coevolution score.
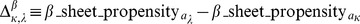
(17)where 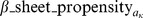 is the value of 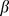 sheet propensity [52] of amino acid 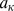.

7. Change of turn propensities accompanied by an amino acid substitution.
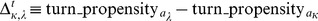
(18)where 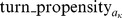 is the value of turn propensity [52] of amino acid 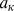.

8. Change of the capability of aromatic interaction accompanied by an amino acid substitution.
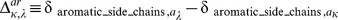
(19)where 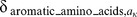 is equal to 1 if 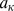 is one of aromatic side-chains (his, phe, trp, and tyr) and 0 otherwise.

9. Change of branched side-chain accompanied by an amino acid substitution.
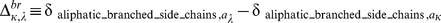
(20)where 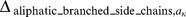 is equal to 1 if 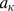 is one of aliphatic branched side-chains (ile, leu and val), and 0 otherwise.

10. Change of cross-link capability accompanied by an amino acid substitution.
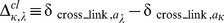
(21)where 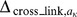 is equal to 1 if 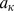 is one of asn, gln, ser and thr, and 0 otherwise.

11. Change of ionic side-chain accompanied by an amino acid substitution.
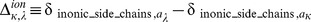
(22)where 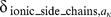 is equal to 1 if 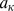 is one of inonic side-chains (asp, glu, arg, and lys), 0.1 if 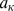 is his, and 0 otherwise.

The correlation coefficients (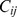) and the partial correlation coefficients (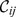) calculated from the feature vectors 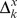 are denoted by 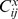 and 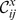, respectively, where 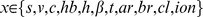.

Dutheil and Galtier [8] employed as 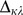 substitution probability, difference of side-chain volume, difference of side-chain charge, difference of side-chain polarity, and Grantham physico-chemical distance [53]. Side-chain polarity as defined by Grantham is essentially the same with hydrophobicity used here. The Grantham physico-chemical distance is a function of volume and polarity, and corresponds to none of quantities used here.

### Protein families and Sequences Used

In order to calculate partial correlation coefficients between sites by taking the inverse of a covariance or correlation matrix, it must be regular so that the dimension of feature vector, which is equal to the total number of branches (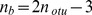, where 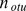 denotes the number of OTUs.) in the present method, must be at least more than or equal to the dimension of the correlation/covariance matrix, which is equal to the number of sites; even if the matrix is singular, partial correlation coefficients may be calculated for some site pairs but not all by using projection operators according to the definition of a partial correlation coefficient in Eq. 11. This restriction is reasonable, because the dimension of feature vector that describes each site must be large enough to distinguish each site from other sites. To obtain statistically reliable numbers, even more sequences than 10 times as many as sites may be needed. In the Pfam database [35], protein domain families consisting of many thousands of homologs (orthologs and paralogs) are included, and each family is expected to be more populated as metagenome projects proceed with next-generation sequencing technologies. Protein domain families used in [16] to infer residue pair couplings in multiple sequence alignments are appropriate to allow us to compare prediction accuracies between the present method and their method. These protein domain families in the Pfam release 26.0 (November 2011) are listed in Table 1. Also, Table 1 shows the Uniprot ID and corresponding protein coordinates (PDB ID) of a target protein in each protein family, for which co-evolving site pairs are predicted.

**Table 1:** Protein families used.

Pfam ID 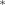	Seed 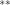		Full 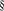		Target protein domain		Fold	#sites
	#seqs	Length	#seqs	Length	Uniprot ID 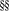	PDB ID 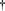	type	/Length 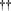
Trans_reg_C	362	114	35180	269	OMPR_ECOLI/156-232	1ODD-A:156-232	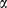	76/77
CH	202	249	5756	650	SPTB2_HUMAN/176-278	1BKR-A:5-107	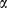	101/103
7tm_1	64	434	26656	2354	OPSD_BOVIN/54-306	1GZM-A:54-306	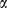 (tm) 	248/253
SH3_1	61	56	8993	210	YES_HUMAN/97-144	2HDA-A:97-144	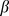	48/48
Cadherin	57	129	18808	494	CADH1_HUMAN/267-366	2O72-A:113-212	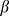	91/100
Trypsin	71	348	14720	1356	TRY2_RAT/24-239	3TGI-E:16-238	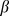	212/216
Kunitz_BPTI	151	81	3090	209	BPT1_BOVIN/39-91	5PTI-A:4-56	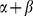	53/53
KH_1	399	104	11484	280	PCBP1_HUMAN/281-343	1WVN-A:7-69	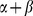	57/63
RRM_1	79	79	31837	580	ELAV4_HUMAN/48-118	1G2E-A:41-111	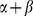	70/71
FKBP_C	174	247	11034	845	O45418_CAEEL/26-118	1R9H-A:26-118	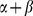	92/93
Lectin_C	44	136	6530	801	CD209_HUMAN/273-379	1SL5-A:273-379	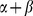	103/107
Thioredoxin	50	123	16281	609	THIO_ALIAC/1-103	1RQM-A:1-103	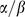	99/103
Response_reg	57	157	103232	804	CHEY_ECOLI/8-121	1E6K-A:8-121	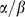	110/114
RNase_H	65	246	13801	574	RNH_ECOLI/2-142	1F21-A:3-142	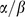	128/140
Ras	61	229	13525	1461	RASH_HUMAN/5-165	5P21-A:5-165	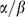	159/161

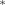
Pfam release 26.0 (November 2011) was used.

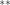
The number of sequences and the length of alignment included in the Pfam seed alignment.

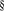
The number of sequences and the length of alignment included in the Pfam full alignment.

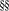
Target protein member in the Pfam family.

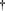
A protein structure corresponding to the target protein domain.

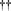
Site positions that are represented by the lower case of characters in Pfam alignments were excluded in the evaluation of prediction accuracy for comparison with the contact prediction published in [16].


 ^‡^Transmembrane 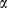.

In the Pfam database, there are two sets of sequence alignments for each protein family; a seed alignment and a full alignment. Also, a phylogenetic tree calculated from each alignment by the FastTree [36] is available. Here the seed alignment and its phylogenetic tree are used to estimate parameters in a mechanistic codon substitution model [37], [38]; refer to the methods section. With those parameters optimized for the seed alignment in the codon-based model, posterior means of characteristic variables at each site in each branch of a phylogenetic tree are estimated for subsets of a full alignment, after branch lengths are optimized.

The full alignments include closely-related sequences whose differences are less than 0.01. The number of branches (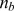) in a phylogenetic tree is proportional to the number of OTUs (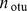) (operational taxonomic units that correspond to sequences in the present case); 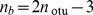 for an unrooted tree. Computational time required for the present calculation increases with increasing number of branches. Including closely-related sequences requires computationally intensive calculation, although it is not much informative; invariant sites do not have any information in the present method, which is designed to detect concurrent and compensatory substitutions between sites in proteins. Thus, subsets made by excluding closely-related sequences from the Pfam full alignments are used in the present calculations. The subsets of a full alignment and their phylogenetic trees are made by removing OTUs that are connected to the parent nodes with branches shorter than a certain threshold (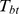), although seed sequences and a target protein are not removed.

In addition, to reduce a computational load in the calculation of the likelihood of a phylogenetic tree, only site positions where amino acids are found in the target protein are extracted from the multiple sequence alignment and used in the present analysis.

Site positions that are represented by the lower case of characters in Pfam alignments were excluded in the evaluation of prediction accuracy for comparison with the contact prediction published in [16].

### A Mechanistic Codon Substitution Model for the Maximum Likelihood Inference of Phylogenetic Tree

A mechanistic codon substitution model, in which each codon substitution rate is proportional to the product of a codon mutation rate and the average fixation probability depending on the type of amino acid replacement, has advantages [37], [38] over nucleotide, amino acid, and empirical codon substitution models in evolutionary analysis of protein-coding sequences, because mutation at the nucleotide level and selection at the amino acid level can be separately evaluated. Even for amino acid sequences of OTUs (operational taxonomic units), the mechanistic codon substitution model with the prior assumption of equal codon usage for them yields smaller AIC values (Akaike Information Criterion) than any amino acid substitution model does (unpublished). Thus, the mechanistic codon substitution model [38] is used here to evaluate the likelihood of a phylogenetic tree and the posterior means of characteristic variables at each site in each branch.

In the mechanistic codon substitution model, in which substitutions are assumed to be in the stationary state of a time-homogeneous reversible Markov process, the substitution probability matrix in time 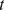 is represented as 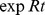 with a substitution rate matrix 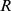, which is defined as.
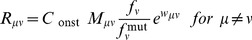
(23)where 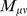 is the mutation rate from codon 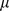 to 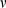, 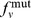 is the equilibrium frequency of codon 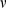 in nucleotide mutations, 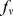 is the equilibrium codon frequency, 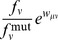 is the average rate of fixation, and 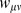 is the selective constraints for mutations from 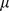 to 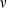; refer to [38] for details. Assuming that nucleotide mutations occur independently at each codon position but multiple nucleotide mutations in a codon can occur in infinitesimal time, the mutation rate matrix 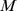 is approximated with 9 parameters; the ratios of nucleotide mutation rates, 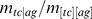, 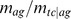, 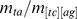, 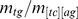, and 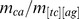, the relative ratio 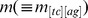 of multiple nucleotide changes, and the equilibrium nucleotide frequencies in nucleotide mutations, 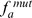, 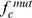, and 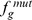. The selective constraint 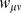 for a protein family is approximated with a linear function of the mean selective constraints that were evaluated [37] by ML-fitting a substitution matrix based on the mechanistic codon model to an empirical amino acid substitution matrix. Here we use the mean selective constraints 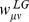 derived from the empirical amino acid substitution matrix LG [54]. The slope 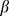 and a constant term 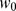 are parameters; 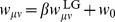. The selective constraint 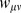 is assumed to vary across sites and the variation of selective constraints [38] has been approximated by a discrete gamma distribution [39] with 4 categories. Thus, one more parameter is a shape parameter 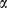 for the discrete gamma distribution. In the result, 12 parameters in addition to the equilibrium frequencies of codons must be determined in this model. See [38] for full details of these parameters.

The equilibrium frequencies of codons are estimated to be equal to codon frequencies in sequences of OTUs with the assumption of equal codon usage for amino acid sequences. Other 12 parameters were estimated by maximizing the likelihood of the Pfam reference tree of Pfam seed sequences. Then, the ML estimates of the parameters obtained from the Pfam seed sequences are used to evaluate branch lengths and posterior means of characteristic variables at each site in each branch of Pfam reference trees for the subsets of Pfam full alignments. Pfam reference trees taken from the Pfam were used for the tree topologies, because optimizing tree topologies for more than a few thousands of sequences require too much computational time. Branch optimization of phylogenetic trees and posterior means of characteristic variables are calculated by using Phyml [55] modified for the mechanistic codon substitution model.

### Definition of Contact Residue Pairs in Protein Structures

Contact residue pairs are arbitrarily defined here as residue pairs whose minimum atomic distances are shorter than 5 Å and which are separated by 6 or more residues along a peptide chain. This definition, especially the latter condition, which was used in Marks et al. [16], is employed here only for the comparison of the present predictions with their predictions of contact residue pairs.

The PDB ID of a protein structure used for a target protein in each Pfam family is listed in Table 1. The amino acid sequences of these PDB entries are just the same as those of the Uniprot IDs, which are also listed in Table 1.

## Results

### Framework of the Present Method

**Figure 1:**
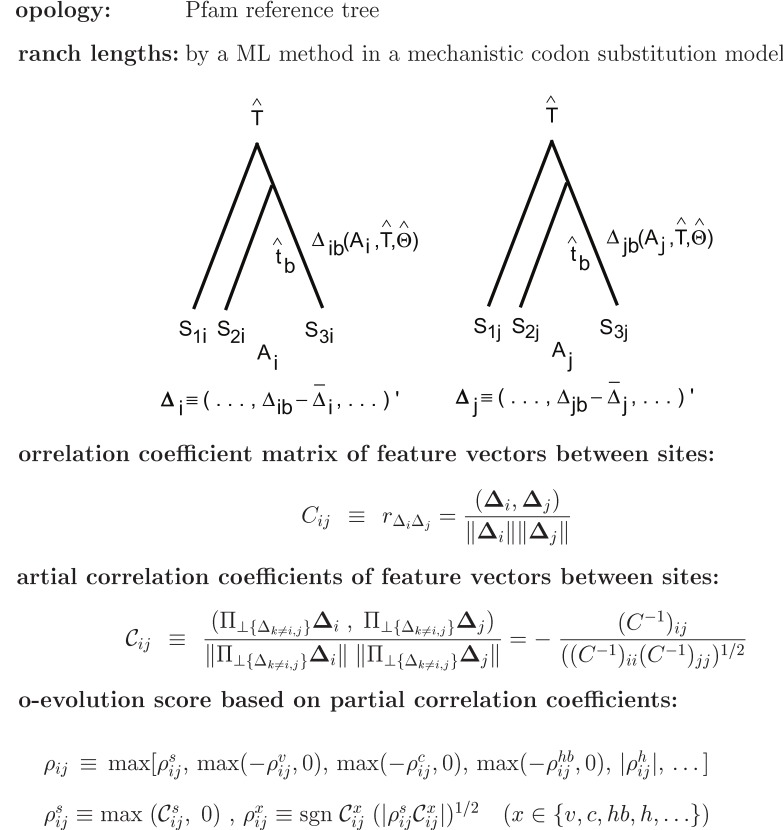
Framework of the present model. See text for details.

The framework of the present method is shown in Fig. 1; refer to the methods sections for details. For each protein family, its phylogenetic tree 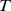 calculated by the FastTree [36] is taken from the Pfam database [35] and branch lengths 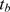 of the tree are optimized by maximizing the likelihood of the tree in a mechanistic codon substitution model [37], [38]. Then, the average changes (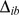) of quantities, which are characteristic of concurrent and compensatory substitutions, accompanied by substitutions at each site 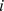 in each branch 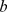 of the phylogenetic tree 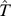 are estimated with the likelihood of each substitution. Their correlation coefficients (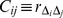) along branches between sites are calculated, and converted to partial correlation coefficients (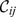), which are correlation coefficients between residual vectors (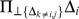 and 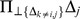) of given two vectors that are perpendicular to a subspace consisting of other vectors except those two vectors (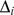 and 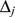) and therefore cannot be accounted for by a linear multiple regression on other vectors. Finally, coevolution scores (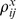) based on the partial correlation coefficients are calculated and an overall coevolution score (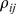) is used to rank site pairs for close spatial proximity.

The following characteristic changes defined by Eqs. 6–7, Eq. 9, and Eqs. 12–22 in the Methods section are used as the feature vector 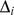 to detect concurrent and compensatory substitutions between sites; (1) occurrence of amino acid substitution: 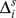, (2) side-chain volume: 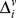, (3) side-chain charge: 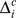, (4) hydrogen-bonding capability: 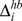, (5) hydrophobicity: 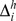, (6) 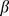 propensity: 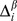, (7) turn propensity: 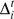, (8) aromatic interaction: 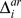, (9) branched side-chain: 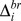, (10) cross-link capability: 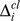, and (11) ionic side-chain: 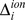. The change of 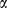 propensity is also examined but not used to define an overall coevolution score. The correlation coefficients (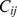), the partial correlation coefficients (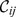), and the coevolution score calculated from the feature vectors 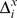 are denoted by 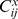, 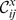, and 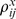, respectively, where 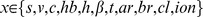.

### Correlation Versus Partial Correlation Coefficients

First, we examined how differently correlation coefficients and partial correlation coefficients of substitution probabilities between sites identify dependent site pairs. The distribution of Pearson's correlation coefficient in the case of no correlation can be well approximated by the Student's 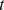 distribution. Therefore, here a correlation coefficient 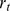 corresponding to the E-value 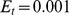 (the P-value 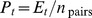) in the Student's t-distribution of the degree of freedom 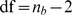 is used as a threshold for significance; where 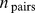 is the number of site pairs and 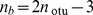 is the number of branches in a unrooted phylogenetic tree.

In Table 2, correlation coefficients (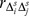) and partial correlation coefficients (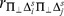) of substitution probabilities along branches between sites are classified into four categories; significantly positive, positive but insignificant, negative but insignificant, significantly negative. In addition, sites pairs in each category are classified according to whether they are contact residue pairs in the protein 3D structure. Contact residue sites are arbitrarily defined as residue pairs whose minimum atomic distances are shorter than 5Å, and which are separated by 6 or more residues along a peptide chain. The upper table shows results for Pearson's correlation coefficients and the lower table does those for partial correlation coefficients. Significantly-positive correlation coefficients are found for almost all site pairs. In the phylogenetic trees of these protein families branch lengths are completely heterogeneous. The expected value of the probability of amino acid substitution in a branch is an increasing function of branch length; 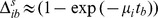 where 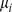 is an amino acid substitution rate for site 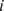. Thus, the Pearson's correlation coefficients of the expected values of substitution probability over branches between sites should be significantly positive, as shown in Table 2. In other words, a main contribution to the correlation coefficients in this case is a phylogenetic correlation, which masks both direct and indirect correlations through other sites; this type of phylogenetic correlation does not exist in the correlation coefficients of physico-chemical changes due to substitutions, because there is no such a simple relationship between the physico-chemical change and branch length. This type of correlation of substitution probability originating from phylogenies can be mostly removed by removing a linear multiple dependence on feature vectors at other sites from the feature vectors at a given site pair, because the expected value of substitution probability in a branch at a site is approximately proportional to the average of substitution probabilities on the branch over sites. Such an operation on feature vectors can also remove indirect correlations through other sites, although only linear multiple dependences on feature vectors at other sites can be removed.

**Table 2:** Correlation (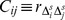) versus partial correlation (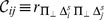) coefficients of concurrent substitutions between sites.

Pfam ID	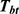 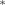	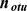 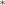	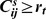 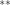		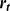 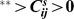		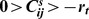 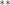		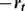 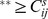	
			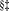	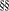	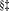	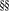	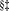	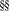	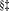	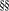
			TP:FP	PPV	TP:FP	PPV	TP:FP	PPV	TP:FP	PPV
Trans_reg_C	0.12	7720	102∶2282	0.04	1∶30	0.03	0∶0	–	0∶0	–
CH	0.01	2960	167∶4226	0.04	2∶73	0.03	0∶2	0.0	0∶0	–
7tm_1	0.1	6302	358∶28576	0.01	0∶0	–	0∶0	–	0∶0	–
SH3_1	0.01	4160	74∶674	0.10	7∶60	0.10	0∶5	0.0	0∶0	–
Cadherin	0.06	7617	214∶3333	0.06	1∶46	0.02	0∶7	0.0	0∶0	–
Trypsin	0.1	6688	617∶20312	0.03	0∶0	–	0∶0	–	0∶0	–
Kunitz_BPTI	0.01	2130	86∶799	0.10	11∶48	0.19	0∶2	0.0	0∶0	–
KH_1	0.01	5114	78∶1116	0.07	1∶41	0.02	0∶4	0.0	0∶0	–
RRM_1	0.15	7684	119∶1839	0.06	0∶0	–	0∶0	–	0∶0	–
FKBP_C	0.01	5695	199∶3445	0.05	0∶10	0.0	0∶1	0.0	0∶0	–
Lectin_C	0.01	4479	234∶4319	0.05	1∶19	0.05	0∶0	–	0∶0	–
Thioredoxin	0.06	7483	188∶4180	0.04	0∶3	0.0	0∶0	–	0∶0	–
Response_reg	0.46	7613	202∶5266	0.04	0∶1	0.0	0∶0	–	0∶0	–
RNase_H	0.01	4782	271∶7152	0.04	0∶5	0.0	0∶0	–	0∶0	–
Ras	0.02	6390	329∶11304	0.03	0∶0	–	0∶0	–	0∶0	–

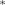
 OTUs connected to their parent nodes with branches shorter than the threshold value 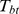 are removed from each Pfam full alignment, and the number of remaining OTUs, 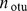, is listed.

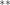
 The 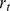 is a threshold for a correlation coefficient corresponding to the E-value 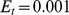 (the P-value 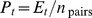) in the Student's t-distribution of the degree of freedom, 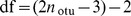, where 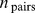 is the number of site pairs, and 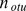 is the number of OTUs.

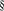
 TP and FP are the numbers of true and false positives, which are the number of contact site pairs and the number of non-contact site pairs predicted as contacts in each category, respectively.

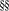
 PPV stands for a positive predictive value; i.e., 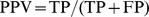.


 ^‡^The numbers of contacts and of sites, and their ratio are listed. Protein structures used to calculate contact residue pairs are listed in Table 1. Neighboring residue pairs within 5 residues (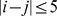) along a peptide chain are excluded in the evaluation of prediction accuracy. Also both terminal sites are excluded from counting in this table.

A partial correlation coefficient defined in Eq. 11 is a correlation coefficient between residuals that cannot be accounted for by a linear multiple regression on the vectors of characteristic changes along branches at other sites. In the case in which dependences on other sites in the variation of substitutions are removed, significantly positive correlations (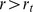) are found only in a limited number of site pairs, and most site pairs show insignificant correlations. Furthermore, site pairs in the category of significantly-positive correlation tend to be contact residue site pairs with significantly-high probabilities; see the column of positive predictive value, PPV 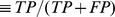, where TP and FP are the numbers of true and false contact residue pairs, respectively.

In Table 3, the fourth and the fifth columns show the PPVs of predictions in which a given number of site pairs are predicted as contacts in the decreasing order of the correlation coefficients or the partial correlation coefficients of substitution probabilities, respectively. The use of the partial correlation coefficients remarkably increase the PPV of contact prediction by removing the phylogenetic and also indirect correlations. These results clearly indicate that the partial correlation coefficients represent the strength of the direct dependences of substitutions between sites.

**Table 3:** Effectiveness of partial correlation coefficients on contact prediction accuracy.

Pfam ID 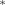	#contacts	TP 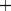 FP 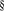	PPV( 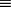 TP/(TP+FP))			
	/#sites 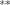		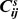 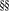	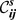 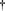	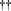	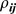 ^‡^ 
Trans_reg_C	103/75	27	0.222	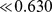	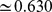	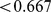
	1.4	37	0.189	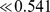	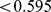	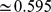
CH	169/100	43	0.047	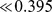	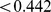	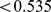
	1.7	57	0.053	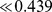	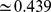	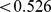
7tm_1	366/247	93	0.011	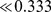	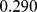	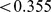
	1.5	124	0.008	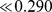	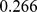	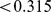
SH3_1	81/46	22	0.227	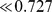	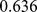	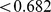
	1.8	29	0.241	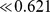	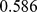	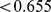
Cadherin	215/90	55	0.291	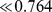	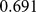	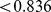
	2.4	73	0.274	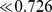	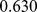	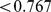
Trypsin	617/210	159	0.396	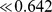	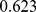	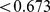
	2.9	212	0.344	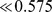	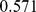	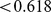
Kunitz_BPTI	105/51	27	0.259	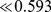	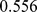	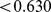
	2.1	37	0.216	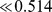	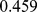	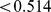
KH_1	79/55	22	0.455	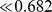	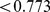	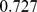
	1.4	30	0.367	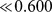	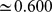	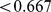
RRM_1	119/68	33	0.273	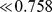	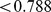	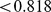
	1.8	44	0.295	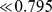	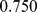	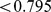
FKBP_C	199/91	50	0.220	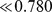	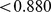	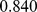
	2.2	66	0.197	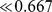	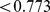	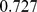
Lectin_C	243/102	61	0.197	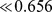	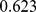	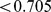
	2.4	82	0.171	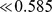	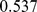	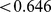
Thioredoxin	188/99	47	0.213	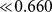	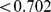	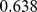
	1.9	62	0.177	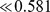	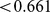	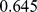
Response_reg	202/110	50	0.000	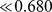	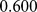	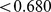
	1.8	67	0.015	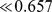	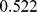	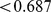
RNase_H	271/127	68	0.162	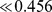	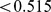	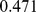
	2.1	91	0.132	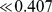	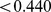	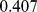
Ras	329/158	83	0.229	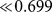	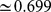	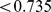
	2.1	111	0.207	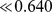	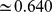	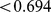

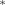
 The threshold 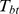 to remove OTUs with short branches and the number 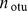 of remaining OTUs that are used for each protein here are listed in Table 2.

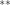
 The numbers of contacts and of sites, and their ratio are listed. Protein structures used to calculate contact residue pairs are listed in Table 1. Neighboring residue pairs within 5 residues (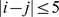) along a peptide chain are excluded in the evaluation of prediction accuracy. Also both terminal sites are excluded from counting in this table.

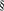
 TP and FP are the numbers of true and false positives, and their sum is equal to the number of predicted contacts; only predictions for 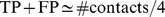 and 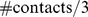 are listed.

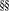
 Correlation coefficients of co-substitution are used as a score.

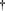
 Partial correlation coefficients of co-substitution are used as a score.

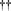
 In Eq. 26 for an overall coevolution score, 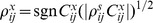 with 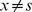 is supposed instead of Eq. 25; in other words, correlation coefficients are used instead of partial correlation coefficients for characteristic changes except co-substitution.


 ^‡^The overall coevolution score defined by Eq. 26 is used.

### Coevolution Score for Site Pairs

Concurrent substitutions between sites require that the direct correlation of substitutions must be positive. Therefore, only positive values of the partial correlation coefficients (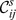) are used to define a coevolution score (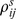) based on concurrent substitutions.
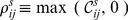
(24)


For all other characteristic variables employed to detect coevolving site pairs, the condition of concurrent substitutions between sites are a premise. Thus, instead of using partial correlations of characteristic variables themselves, the geometric mean of the partial correlation coefficient of each characteristic variable and the coevolution score based on concurrent substitutions is used as a coevolution score based on each characteristic change.
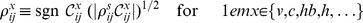
(25)


As mentioned in the Method section, negative correlations are required for characteristic variables such as volume, charge, and hydrogen bonding capacity to reflect compensatory substitutions. In Table 4, TP and FP over all 15 protein families listed in Table 2 for each category of significantly positive (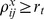) and negative (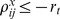) correlations under the condition of 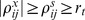 are listed for each characteristic variable. In the cases of volume, charge, and hydrogen bonding capacity, PPV for contact residue pairs is clearly larger in the category of significantly negative correlation than significantly positive correlation, indicating that these quantities to detect compensatory substitutions between sites are good predictors for close spatial proximity. Besides, there are more site pairs with significantly negative correlations than with significantly positive correlations, clearly indicating the presence of structural constraints against these physico-chemical changes.

**Table 4:** Coevolution score (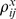) based on each characteristic variable.

Characteristic	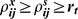 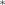			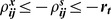 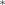		
variable	TP 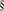	FP 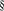	PPV 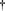	TP 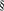	FP 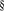	PPV 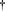
	over all protein families					
Substitution	687	642	0.52			
Volume	18	20	0.47	73	10	**0.88** ^‡^ 
Charge	6	8	0.43	134	54	**0.71** ^‡^ 
Hydrogen bond	4	11	0.27	125	51	**0.71** ^‡^ 
Hydrophobicity	23	13	**0.64** ^‡^ 	23	16	**0.59** ^‡^ 
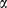 propensity	14	20	0.41	9	10	0.47
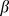 propensity	24	17	**0.59** ^‡^ 	30	14	**0.68** ^‡^ 
Turn propensity	21	18	**0.54** ^‡^ 	17	15	**0.53** ^‡^ 
Aromatic interaction	30	10	**0.75** ^‡^ 	16	14	**0.53** ^‡^ 
Branched side-chain	26	16	**0.62** ^‡^ 	20	8	**0.71** ^‡^ 
Cross link	23	12	**0.66** ^‡^ 	5	9	0.36
Ionic side-chain	27	15	**0.64** ^‡^ 	14	18	0.44

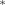
 See Eqs. 24 and 25 for the definitions of 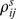 and 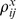, respectively. The 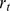 is a threshold for a correlation coefficient corresponding to the E-value 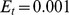 (the P-value 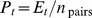) in the Student's t-distribution of the degree of freedom, 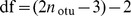, where 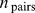 is the number of site pairs, and 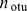 is the number of OTUs.

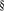
 TP and FP are the numbers of true and false contact residue pairs over all 15 protein families listed in Table 2; protein structures used to calculate contact residue pairs are listed in Table 1. Neighboring residue pairs within 5 residues (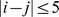) along a peptide chain are excluded in the evaluation of prediction accuracy. Also both terminal sites are excluded from counting in this table.

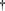
 PPV stands for a positive predictive value; i.e., 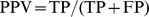.


 ^‡^These PPVs are larger than the PPV for concurrent substitutions, i.e., 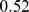 for 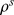.

To improve contact prediction by using characteristic variables 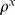 together with the characteristic variable 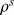 of concurrent substitutions, the PPV for the category of significantly positive or negative correlations should be larger than the PPV for concurrent substitutions. Both categories of significantly positive and negative correlations show better PPVs in the characteristic variables of hydrophobicity, 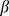 and turn propensities, aromatic interaction and branched side-chain. In the characteristic variables of cross-link capability and ionic side-chain, only the category of significantly positive correlation shows better PPV than the category of significantly positive correlation for concurrent substitutions. The 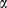 propensity is not effective to detect contact residue pairs, although it may be effective to detect residue pairs within a helix or within helices. Based on these results, an overall coevolution score for site pair (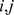) is defined here as.
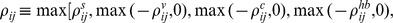


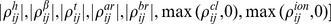
(26)


In Table 3, the different effects of the correlation and the partial correlation coefficients of the characteristic variables other than substitution probability on contact prediction accuracy are shown in the sixth and seventh columns, respectively. The PPVs shown in the seventh column, for which a given number of site pairs are predicted as contacts in the decreasing order of the overall coevolution score defined by Eq. 26 with Eq. 25, are mostly better than the PPVs in the sixth column, for which 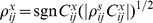 with 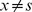 is supposed instead of Eq. 25. This result indicates that indirect correlations through other residues can be reduced by the use of partial correlation coefficient.

### Contact Prediction based on the Overall Coevolution Score 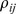


Coevolving sites pairs are selected for contacts in the decreasing order of the overall coevolution score 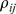. Although this score for coevolution appears to be able to predict contact site pairs, preliminary results of contact prediction indicate that both terminal sites in multiple sequence alignments often have large values of 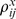 (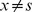) for any other site, and also that there are a few sites showing extremely large values for 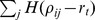; the 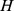 denotes the Heaviside step function. Such an anomalous feature may indicates a poor quality at these sites in multiple sequence alignments. Although a method for the assessment of alignment confidence was proposed [56], the following simple strategy for terminal sites is employed here.

1. the coevolution scores of 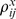 (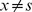) are ignored for both terminal sites in multiple sequence alignments; that is, 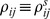.
2. Also, if 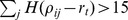, 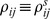 will be used for site 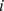, and
3. if 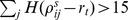, 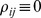 will be used and such a site will be excluded in contact prediction.

The threshold value 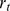 used here is the value of correlation coefficient corresponding to 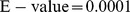 in the Student's t-distribution. The threshold number of contacts per residue, 15, is appropriate for the present case in which 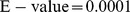 and residue pairs separated by 5 or fewer positions in a sequence are excluded in the present predictions for comparison with those based on the DI score [16]. In the present contact predictions, only one site that is the N-terminal site in the multiple sequence alignment for the KH_1 was excluded as an anomalous site.

Needless to say, the norm of any characteristic change vector is almost zero for invariant sites; 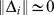. Therefore, invariant sites are excluded from contact prediction in the present method.

The coevolution scores, the overall coevolution score and rank of each site pair in each protein are listed in text files provided as Data S1.

### Contribution of Each Coevolution Score, 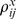, on Contact Prediction

**Figure 2:**
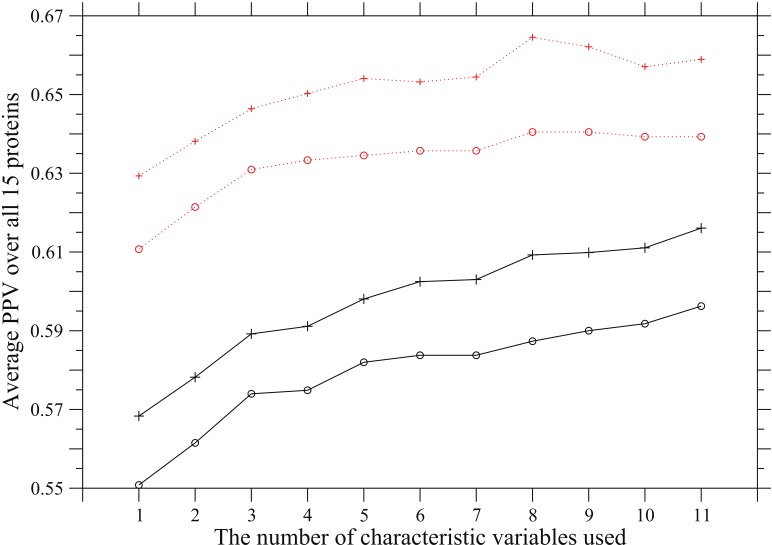
Dependence of PPV on the number of characteristic variables used. Average PPVs are plotted against the number of characteristic variables used to score co-substitutions between sites. The characteristic variables except 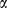 propensity listed in Table 4 are added in the listed order to define an overall coevolution score; that is, (1) occurrence of amino acid substitution, (2) side-chain volume, (3) charge, (4) hydrogen-bonding capability, (5) hydrophobicity, (6) 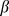 and (7) turn propensities, (8) aromatic interaction, (9) branched side-chain, (10) cross-link capability, and (11) ionic side-chain. The solid and dotted lines correspond to predictions in which the ratio of the predicted to the true contacts is equal to 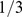 or 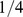, respectively. The plus marks and open circles show the averages of PPV over all 15 proteins and the values of 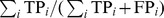, where the sum is taken over all 15 proteins.

Contribution of each coevolution score, 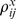, on contact prediction is shown in Fig. 2, in which average PPVs over all 15 proteins are plotted against the number of characteristic variables used to define an overall coevolution score. The solid and dotted lines correspond to predictions in which the ratio of the predicted to the true contacts is equal to 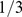 or 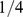, respectively. The plus marks and open circles show the averages of PPV over all 15 proteins and the values of 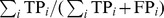, respectively, where the sum is taken over all 15 proteins. The characteristic variables except 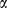 propensity listed in Table 4 are added in the listed order to define an overall coevolution score in Eq. 26; that is, (1) occurrence of amino acid substitution, (2) side-chain volume, (3) charge, (4) hydrogen-bonding capability, (5) hydrophobicity, (6) 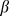 and (7) turn propensities, (8) aromatic interaction, (9) branched side-chain, (10) cross-link capability, and (11) ionic side-chain. The dependence of PPV on the number of characteristic variables used for each protein are shown in Fig. S1. These figures show that in average the prediction accuracy of contact site pairs is improved by adding the characteristic variables in the order above, although the prediction accuracy of each protein is not always improved, and the average increments of prediction accuracy by adding the characteristic variables one by one are not large.

### Accuracy of Contact Site Pairs Predicted on the basis of the Overall Coevolution Score

Accuracies of predictions based on the overall coevolution score and on the direct information (DI) score [16] calculated by a maximum entropy model, which was shown to be more accurate than other prediction methods (mutual information, statistical coupling analysis [9], [13], and Bayesian network model [14] ), are compared by using three measures in Table 5 for protein families listed in Table 1; the predictions based on the DI are taken from http://cbio.mskcc.org/foldingproteins/Appendix_A1. Those three measures are PPV, mean Euclidean distance from predicted site pairs to the nearest true contact (MDPNT) in the 2-dimensional sequence-position space, and the mean Euclidean distance from every true contact to the nearest predicted site pair (MDTNP). The MDPNT and MDTNP, which were defined and used in [16], are qualitative measures of false positives and of the spread of predicted site pairs over true contacts, respectively. Smaller values of these measures indicate better predictions.

**Table 5:** Accuracy of contact prediction based on the overall coevolution score (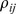).

Pfam ID 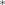	#contacts	TP 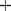 FP 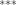	PPV 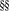 ^‡^		PPV 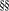 ^‡‡^		MDPNT 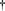 ^‡‡^		MDTNP 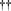 ^‡‡^	
	/#sites 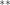		DI 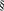	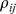	DI 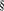	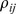	DI 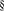	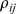	DI 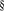	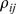
Trans_reg_C	111/76	27	0.556	**0.667**	0.556	**0.667**	1.30	**0.94**	4.20	**3.28**
	1.5	37	0.459	**0.622**	0.432	**0.622**	1.72	**1.16**	3.64	**2.82**
CH	172/101	43	0.535	**0.558**	**0.488**	0.465	**2.23**	2.55	4.59	**4.37**
	1.7	57	0.456	**0.561**	0.439	**0.491**	**2.12**	2.44	3.70	**3.30**
7tm_1	372/248	93	0.290	**0.409**	0.194	**0.344**	7.43	**5.31**	12.68	**7.71**
	1.5	124	0.282	**0.355**	0.169	**0.306**	7.30	**5.33**	12.18	**6.40**
SH3_1	89/48	22	0.636	**0.682**	0.636	**0.682**	0.83	**0.51**	**1.69**	2.34
	1.9	29	0.552	**0.655**	0.552	**0.655**	1.15	**0.62**	1.56	**1.51**
Cadherin	220/91	55	0.836	0.836	0.818	**0.836**	0.59	**0.25**	1.98	1.98
	2.4	73	0.753	**0.767**	0.753	**0.767**	0.64	**0.45**	1.60	1.60
Trypsin	636/212	159	0.642	**0.692**	0.591	**0.673**	1.75	**1.20**	3.26	**3.10**
	3.0	212	0.580	**0.627**	0.533	**0.613**	2.26	**1.65**	2.83	**1.94**
Kunitz_BPTI	111/53	27	0.593	**0.630**	0.444	**0.593**	1.40	**1.18**	2.31	**2.08**
	2.1	37	**0.649**	0.486	**0.541**	0.486	**1.13**	1.46	**1.86**	1.94
KH_1	90/57	22	0.545	**0.773**	0.500	**0.773**	0.99	**0.51**	**2.41**	3.29
	1.6	30	0.533	**0.733**	0.533	**0.700**	1.07	**0.56**	**2.16**	3.05
RRM_1	133/70	33	0.788	**0.818**	0.758	**0.818**	**0.52**	0.55	2.86	**2.36**
	1.9	44	0.750	**0.795**	0.705	**0.795**	0.83	**0.49**	2.49	**1.84**
FKBP_C	200/92	50	0.760	**0.840**	0.760	**0.840**	**0.53**	0.69	1.97	**1.85**
	2.2	66	0.712	**0.727**	0.697	**0.727**	0.94	**0.85**	1.66	**1.51**
Lectin_C	246/103	61	**0.803**	0.721	**0.770**	0.705	**0.80**	0.94	2.93	**2.67**
	2.4	82	**0.683**	0.659	**0.671**	0.646	1.19	**1.17**	2.54	**2.32**
Thioredoxin	188/99	47	0.532	**0.681**	0.532	**0.638**	0.98	**0.85**	3.43	**2.33**
	1.9	62	0.597	**0.661**	0.565	**0.645**	0.94	**0.91**	3.16	**1.86**
Response_reg	202/110	50	0.680	**0.700**	0.660	**0.680**	**0.86**	0.88	3.39	**3.06**
	1.8	67	0.657	**0.701**	0.642	**0.687**	1.01	**0.92**	2.54	**2.29**
RNase_H	273/128	68	**0.588**	0.471	**0.559**	0.471	**1.51**	1.53	**3.61**	5.44
	2.1	91	**0.571**	0.407	**0.549**	0.407	**1.55**	2.19	3.27	**3.07**
Ras	335/159	83	0.699	0.699	0.699	0.699	**0.94**	1.05	**2.98**	3.68
	2.1	111	0.640	**0.694**	0.631	**0.685**	**1.12**	1.45	**2.40**	2.51

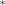
 The threshold 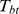 to remove OTUs with short branches and the number 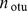 of remaining OTUs that are used for each protein here are listed in Table 2.

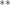
 The numbers of contacts and of sites, and their ratio are listed. Protein structures used to calculate contact residue pairs are listed in Table 1. Neighboring residue pairs within 5 residues (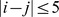) along a peptide chain are excluded in the evaluation of prediction accuracy.

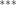
 TP and FP are the numbers of true and false positives, and their sum is equal to the number of predicted contacts; only predictions for 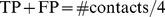 and 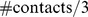 are listed.

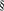
 DI means the prediction based on the direct information (DI) score published in [16].

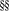
 PPV stands for a positive predictive value; i.e., 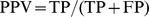. Better values are typed in a bold font.

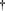
 MDPNT stands for the mean Euclidean distance from predicted site pairs to the nearest true contact in the 2-dimensional sequence-position space [16]. Better values are typed in a bold font.

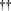
 MDTNP stands for the mean Euclidean distance from every true contact to the nearest predicted site pair in the 2-dimensional sequence-position space [16]. Better values are typed in a bold font.


 ^‡^Filters that are based on a secondary structure prediction and cysteine pairs, and were applied to DI in [16], are applied to both DI and 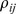. For DI, an additional filter [16] based on residue conservation is also used.


 ^‡‡^Only the conservation filter is used for DI but no filter is used for 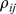.

The filter [16] based on residue conservation degree is applied for all DI-based predictions referred to in the present manuscript; that is, sites where more than 95% of sequences have the dominant residue except cysteine are excluded from contact prediction; refer to Text S1 of [16]. Invariant sites are excluded from contact prediction in the present method, too. In addition, for the predictions listed in the fourth and the fifth columns, which are based on the DI score or on the present coevolution score, the filters that are based on a secondary structure prediction and on cysteine-cysteine pairs for the DI-based contact prediction are applied; refer to Text S1 of [16], In other words, contact residue pairs that conflict with a predicted secondary structure are not allowed, and multiple cysteine-cysteine contacts are not allowed for cysteine residues.

**Figure 3:**
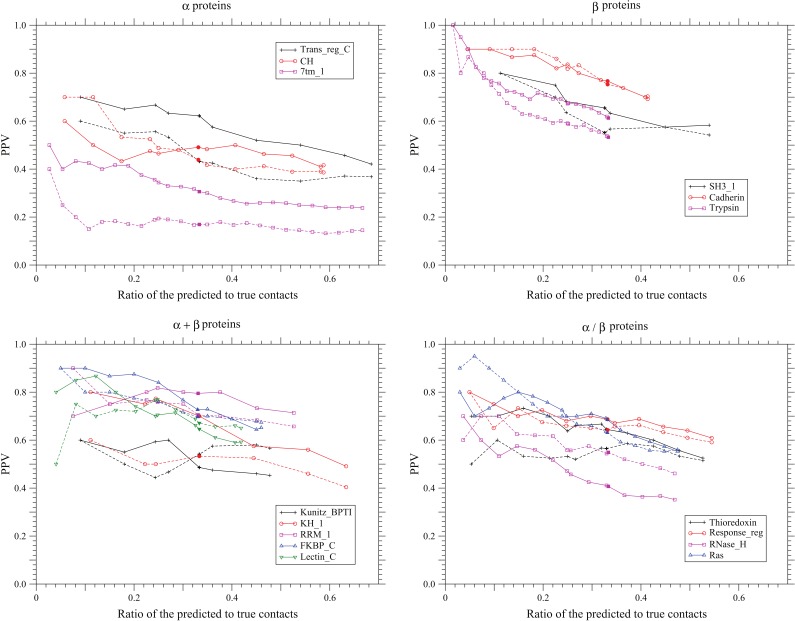
Dependence of PPV on the number of predicted contacts. The dependences of the positive predictive values on the total number of predicted contacts are shown for each protein fold of 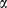, 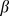, 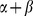, and 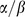. The solid and dotted lines show the PPVs of the present method and the method based on the DI score [16], respectively. Only the conservation filter [16] is applied for the DI score. The total number of predicted site pairs is shown in the scale of the ratio of the number of predicted site pairs to the number of true contacts. The total number of predicted site pairs takes every 10 from 10 to a sequence length; also PPVs for the numbers of predicted site pairs equal to one fourth or one third of true contacts are plotted. The filled marks indicate the points corresponding to the number of predicted site pairs equal to one third of the number of true contacts. The number of sequences used here for each protein family is one listed in Table 1.

The accuracy of three-dimensional structure prediction based on inferred distance constraints will depend on false positive rate and also fold type. The reliability of predicted coevolving site pairs decreases with decreasing value of coevolution score, and coevolving site pairs are selected in the decreasing order of coevolution score. Therefore, prediction accuracy tends to decrease as the total number of predicted sites pairs increases; see Fig. 3. It was reported [57], [58] that the quality of predicted 3D structure depends on the accuracy of inferred contacts more than missing contacts. In Table 5, the results of predictions in which the numbers of predicted contacts are equal to one fourth or one third of the number of true contacts are listed for each protein family. Although the number of contacts well scales with the chain length [59], the ratio of the number of contacts to the chain length somewhat varies depending on proteins as shown in Table 5. One third of the total number of true contacts is equal to the sequence length in the case of Trypsin, which has the largest number, 3.0, of contacts per residue in Table 5, and equal to half of the sequence length in the case of Trans_reg_c and 7tm_1, which have the smallest number, 1.5, of contacts per residue. These ratios, 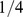 and 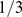, were chosen, because for the same set of protein domain families, it was reported [16] that one needs about 0.5 to 0.75 predicted distance constraints per residue, which correspond to about 0.25 to 0.35 of the total number of contacts, to achieve reasonable three-dimensional structure prediction. This result is consistent with other reports in which three-dimensional structures were reconstructed [57] from predicted contact maps or essential contacts determining protein structure were computed [60].

**Figure 4:**
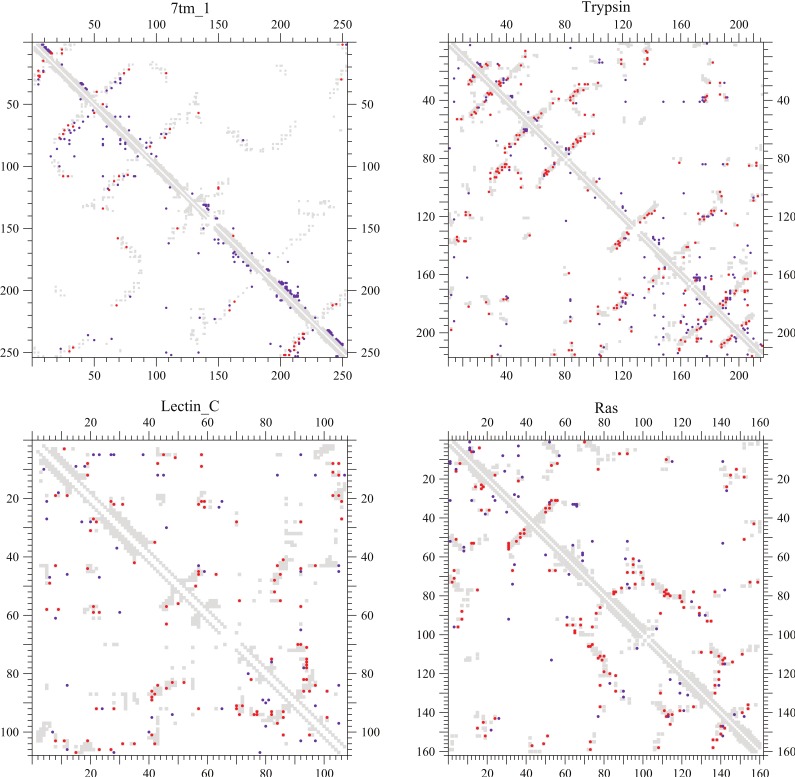
Coevolving site pairs versus DI residue pairs. Residue pairs whose minimum atomic distances are shorter than 5 Å in a protein structure and coevolving site pairs predicted are shown by gray filled-squares and by red or indigo filled-circles in the lower-left half of each figure, respectively. For comparison, such residue-residue proximities and predicted contact residue pairs with high DI scores in [16] are also shown by gray filled-squares and by red or indigo filled-circles in the upper-right half of each figure, respectively; only the conservation filter is applied but the filters based on a secondary structure prediction and for cysteine pairs are not applied to the DI scores. Red and indigo filled-circles correspond to true and false contact residue pairs, respectively. Residue pairs separated by five or fewer positions (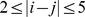) in a sequence may be shown with the gray filled-squares but are excluded as well as nearest neighbors in both the predictions. The total numbers of coevolving site pairs and DI residue pairs plotted for each protein are both equal to one third of true contacts (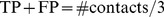). The PPVs of both the methods for each protein are listed in Table 5.

In Fig. 4 and Fig. S2, coevolving site pairs are shown in the lower half of each triangular map in comparison with residue pairs whose minimum atomic distances are less than 5 Å. For comparison, contact residue pairs predicted with high DI scores [16] are also shown in the upper half of each triangular map. Gray filled-squares, red and indigo filled-circles indicate such residue-residue proximities, true and false positives in contact prediction, respectively. It should be noted here that residue pairs separated by 5 or fewer positions (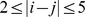) in a sequence may be shown with the gray filled-squares but are excluded as well as nearest neighbors in both the prediction of coevolving site pairs and the contact prediction with the DI score. The total number of predicted site pairs is equal to one third of the total number of true contacts in each protein structure.

In Table 5, which method is better in the accuracy of contact prediction is indicated by a bold font. The PPVs of the present method are comparable to those of the DI method for most of the proteins irrespective of the use of the filters based on predicted secondary structures and on cysteine-cysteine pairs. The use of those filters improves the PPVs of the DI and the present methods at most by about 10–15% for 7tm_1 and Kunitz_BPTI and by about 10% for CH, respectively. However, for most of other proteins, the improvements of the PPVs of the DI and the present methods are less than 5%, although this fact does not necessarily indicate that both the predictions are almost compatible with the secondary structure predictions.

In Fig. 3, the PPVs of the present method and the DI method are drawn by solid and dotted lines as a function of the ratio of predicted to true contacts, respectively. Also, the values of MDPNT and MDTNP are compared between the present and DI methods and also between the protein families in Figs. S3 and S4, respectively. The good performance of the present method is shown over a wide range of predicted site pairs.

### Dependence of the Prediction Accuracy on the Number of Predicted Site Pairs

The dependences of the accuracy of predicted contacts on their number are shown in Fig. 3 for PPV, in Fig. S3 for MDPNT, and in Fig. S4 for MDTNP. The total number of predicted site pairs takes every 10 from 10 to a sequence length; also accuracies for the numbers of predicted contacts equal to one third or one fourth of true contacts are plotted. Here, in order to compare prediction accuracies between protein families, the total number of predicted contacts is shown in the scale of the ratio of predicted to true contacts. It is clearly shown that there is an overall trend for PPV to decrease monotonically as increasing number of predicted site pairs. However, exceptional increases of PPV are also observed with increasing number of site pairs predicted. In the protein family of CH, PPV changes from 0.43 to 0.5 as the number of predicted site pairs increases from 30 to 50. Because except the case of CH such abnormal increases of PPV often occur in the range of small numbers of predicted site pairs, i.e., from 10 to 30, they may be caused by statistical fluctuations.

It is shown in Table 5 and Fig. S3 that the relationships of MDPNT with the ratio of predicted to true contacts are almost inverse of that of PPV, indicating that the MDPNT and PPV are two different measures of the quality of predicted site pairs but result in similar evaluations. On the other hand, MDTNP, which measures the spread of predicted site pairs over true contacts, measures the qualities of predicted contacts differently from PPV and MDPNT. It tends to decrease monotonically as increasing number of predicted site pairs irrespective of the quality of prediction accuracy, and therefore it is not appropriate to measure the dependence of prediction accuracy on the total number of predicted site pairs.

### Dependence of the Prediction Accuracy on Protein Fold Types

As expected, prediction accuracy is different between proteins. However, it is unexpected that prediction accuracy may be slightly lower for 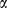 proteins, at least for the present three proteins, than for 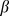 proteins; see Fig. 3. Especially the prediction accuracy for the membrane protein 7tm_1 is remarkably lower than other two 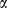 proteins. This feature is observed in both the present and the DI methods. Thus, this feature may originate in differences between structural constraints in 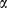-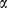 packing and in the packings of 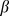 strands and 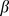 sheets, although the low prediction accuracy for the membrane protein 7tm_1 would result from 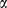-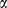 packing peculiar to membrane proteins. Here it should be noted that the 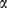 proteins have less contacts per residue than the 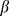 proteins; see Table 5. A definitive answer must be postponed until more 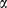 proteins are analyzed.

### Dependence of the Prediction Accuracy on the Diversity and the Number of Sequences Used

**Figure 5:**
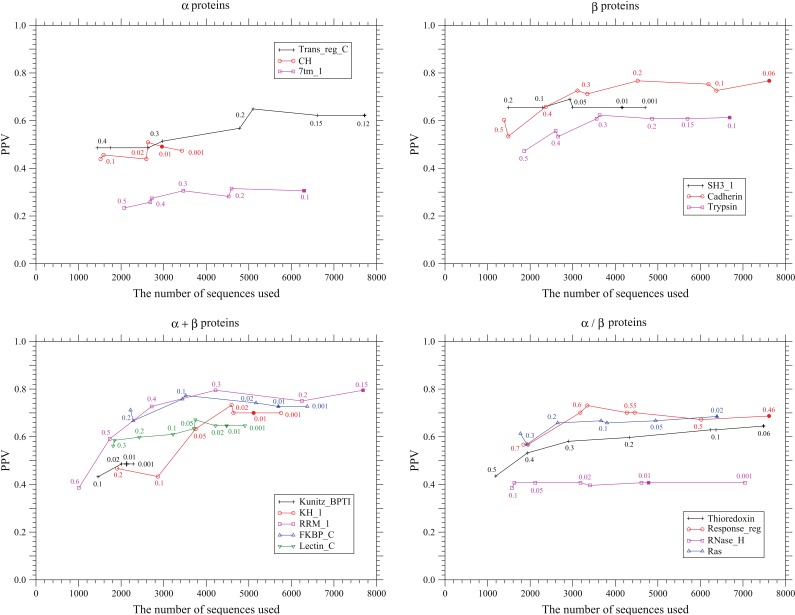
Dependence of PPV on the number of sequences used. The positive predictive values are plotted against the total number of homologous sequences used for each prediction. The total numbers of coevolving site pairs predicted for each protein are equal to one third of true contacts. The filled marks indicate the points corresponding to the number of used sequences listed for each protein family in Table 1. The values written near each data point indicate the threshold value 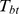; OTUs connected to their parent nodes with branches shorter than this threshold value are removed in the Pfam reference tree of the Pfam full sequences used for each prediction. Some data points correspond to datasets generated by using the same value of the threshold but by removing different OTUs.

Multiple subsets of a full alignment are generated by using different values of threshold 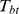 for branch length to remove OTUs connected to their parent nodes with short branches in the Pfam reference tree. In Fig. 5, S5, and S6, the PPVs, MDPNTs, and MDTNPs calculated from each data are plotted against the number of sequences used, respectively. Because the threshold values used to generate each dataset should also affect the accuracy of prediction, they are written near each data point. A general tendency is of course that the PPV and MDPNT are improved by using more sequences. However, the number of sequences and the threshold 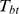 where accuracy improvement is saturated are very different between protein families. For example, in the case of SH3_1, no significant improvement in the PPV and MDPNT is observed in a wide range of 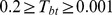, even if the number of sequences increases from 1500 to 4000. In RNase_H, the PPV and MDPNT are almost constant in the range of 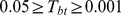 and 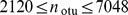. In Response_reg, after the PPV reaches the highest value 0.73 at 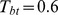 and 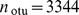, it even decreases to 0.69 in 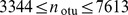, although its decrement is not large and the MDPNT is almost constant in this region. Multiple sequence alignments may include many sites where significant fraction of sequences have deletions, reducing effectively the number of sequences; for example, in the case of RNase_H. However, it may be worth increasing the number of sequences until 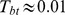; the threshold will be 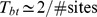, which is a condition for one co-substitution (two substitutions) to occur in a sequence at the branch. Here calculations have been carried out until 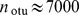 or 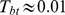. Sequences more than a thousand are necessary to get a reliable prediction for proteins consisting of a few hundred residues.

Some data points in Fig. 5, S6, and S5 correspond to datasets generated by using the same value of threshold but by removing different OTUs. PPV often differs between such datasets, although the difference of PPV ranges from a few percent to 8 percent; see the PPVs for 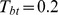 of Trans_ref_C, 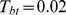 of CH, and 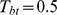 of Cadherin in Fig. 5. This fact indicates that the distribution of sequences in a sequence space significantly affects prediction accuracy. Also, it is indicated that some site pairs predicted are still based on rare events of concurrent substitutions in a tree.

## Discussion

### Partial Correlation Coefficients Effectively Extract Direct Correlations between Sites

The present method is based on co-substitutions between sites. As shown in Table 2–3, Pearson's correlation coefficients of substitution probabilities between sites over branches of a phylogenetic tree reflect phylogenetic correlations, which originate from a fact that at any site substitution probability in a branch is an increasing function of branch length. This type of phylogenetic correlations are specific to substitution probabilities along branches between sites, but do not exit between other characteristic variables used here. In order to detect concurrent substitutions, such phylogenetic correlations must be removed. Substitution probabilities in each branch at sites may be corrected by using the branch length. However, the estimation of branch length is model-dependent. Here, instead substitution probabilities in each branch at a given pair of sites were corrected by removing a linear multiple dependence on substitution vectors at other sites, and then their correlation coefficients, which are named as partial correlation coefficients, were calculated. This correction is justified, because the expected value of substitution probability in a branch at a site is approximately proportional to the average of substitution probabilities on the branch over sites. In addition, this correction on feature vectors can remove indirect correlations through other sites, although only linear multiple dependences on feature vectors at other sites can be removed. It was shown in Table 3 that the partial correlation coefficients of substitution probabilities between sites over branches can well detect co-substitutions, and indirect correlations of any feature vector through other sites can be reduced as well.

### Excellent Prediction Accuracy of Residue-residue Proximity

Here, with respect to the prediction accuracy of contact residue pairs the present method has been shown to be comparable to the DI method [15], [16] that seems to be one of the best methods, in a range of the total number of predicted coevolving site pairs from one fourth of sequence length to sequence length, for 15 protein families of the four major SCOP fold classes and of short to long sequence. Although prediction accuracy is insensitive to sequence length, it is slightly lower for 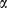 proteins than for 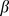 proteins, reflecting differences between 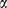
–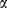 and 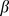
–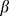 packings; especially prediction accuracy for a membrane protein 7tm_1 is significantly lower than for other proteins. Overall, the prediction accuracy of the present method is comparable to that by the joint distribution of amino acid types between sites in a multiple sequence alignment, which was shown to be sufficient to achieve reasonable three-dimensional structure prediction [16]; for a membrane protein 7tm_1, the present method showed better prediction accuracy.

Prediction accuracy of contact residue pairs is different between the protein families. Possible reasons for false positives include (1) statistical noise due to an insufficient number of sequences, insufficient diversity of sequences, and incorrect matches in a multiple sequence alignment and an incorrect phylogenetic tree, (2) structural and functional constraints from other residues, which are not taken into account in the calculation of partial correlation coefficients from a correlation matrix, within a protein or even through other molecules involved in a molecular complex such as oligomerization, protein-substrate, and protein-DNA, (3) structural variance in homologous proteins, although each Pfam family is assumed to be iso-structural. Especially, for proteins whose functional states are homomeric, inter-residue and intra-residue contacts must be discriminated.

Of course prediction accuracy depends on the size of sequences used and their diversity. A general trend is that prediction accuracy becomes better as increasing number of sequences used, although the diversity of sequences in protein families is more effective than the number of sequences itself. Also, the presence of many deletions in sequences reduces the value of including those sequences. The present subset (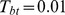) of the full alignment of RNase_H family consists of more than 4700 sequences, but their multiple sequence alignment includes many sites where the significant fraction of sequences have deletions.

Here, branch lengths of OTUs (sequences) from their parent nodes in a phylogenetic tree are used to get less sequences but as diverse sequences as possible. The maximum number of sequences to be tried for the present method will correspond to a dataset generated by 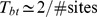, which is a condition for one co-substitution (two substitutions) to occur in a sequence at the branch. However, the cost-effective number of sequences to be used is different between protein families, indicating that the distribution of sequences in a sequence space significantly affects prediction accuracy. At this stage, we could not find a general rule for the cost-effective number of sequences to be used.

In order to get useful numbers (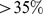) of PPV, more than 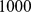 sequences whose branch lengths from their parent nodes are longer than 0.01 amino acid substitutions per site would be needed. This requirement seems to be similar to that for the maximum entropy model [16], in which the order of one thousand sequences are required to reduce statistical noises including phylogenetic bias in frequency counts.

### Dependences on the Accuracies of a Substitution Model, Tree Topology, and a Sequence Alignment

In the present evaluation of characteristic variables at each site in each branch, a mechanistic codon model with equal codon usage is used because even for amino acid sequences it yields smaller AIC values than any amino acid substitution model does. However, amino acid substitution models may be used instead, because smaller AIC values do not necessarily mean that the detection of coevolving positions is improved; substitution mapping on phylogenies was shown to be robust to the input model [47].

In order to examine the dependence of prediction accuracy on tree topology, phylogenetic trees optimized by an approximate ML method, FastTree2 with the default option (JTT and CAT) [61], for datasets of 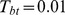 or full alignments have been used as tree topologies instead of the Pfam reference trees for the protein alignments whose 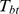 values are listed in Table 2. Also, phylogenetic trees optimized by a maximum-likelihood method ExaML [62] with the default option (JTT and PSR) following the FastTree2 have been used for CH, SH3_1, Kunitz_BPTI, and RNase_H. The accuracies of predictions using those optimized trees are compared with those using the Pfam reference trees in Table S1. This table also shows the log-likelihood value of a tree with branch lengths optimized in a codon model for each tree topology. The prediction accuracy of contact site pairs were not significantly improved in the optimized tree topologies; the PPV could be improved at most by a few percent but could be even worse. The variation of the PPV values was almost in the range of those between datasets generated by using the same value of threshold (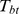) but by removing different OTUs. These results may indicates that the effectiveness of optimization of tree topology is limited due to the accuracy of a sequence alignment. This indication is consistent with a report [63] that the likelihood maximization of tree topology by the RAxML [62] was not effective in comparison with the FastTree [36] in estimating correct topologies with less accurate DNA alignments, which might be estimated on very large datasets.

An accurate multiple sequence alignment will be critical to increase prediction accuracy, because phylogenetic inference for co-substitutions as well as tree topology is based on alignments. In the present calculations, sites that correspond to deletions in a target protein structure are excluded in the optimization of tree branches and in the calculation of partial correlation coefficients. The calculation of partial correlation coefficients by including those sites has been attempted for the Kunitz_BPTI and RNase_H domain families. No improvement was obtained at least for these protein families.

### Significance of Compensatory Substitutions in Protein Evolution

It has been shown that site pairs giving the significant values of partial correlation coefficients for substitutions, which concurrently occurred in branches of a phylogenetic tree and would be mostly compensatory substitutions, well correspond to contact site pairs in protein 3D structures. In compensatory substitutions, the fitness of first mutations must be negative, and successive mutations must occur to compensate the negative effect of the first mutation. A time scale in which compensatory mutations successively occur is much shorter than the time scale of protein evolution that is the order of fixation time for neutral mutations, otherwise negative mutants will be eliminated from a gene pool by selection. Thus, negative substitutions and their compensatory substitutions are expected to be observed as concurrent substitutions in the same branch of a tree. If substitutions are completely neutral, there will be no correlation in time when substitutions occur. Thus, a fact that contact site pairs can be well predicted by the present method indicates that compensatory substitutions are significant in protein evolution. Significance of compensatory substitutions was also indicated by a fact that likelihoods of phylogenetic trees can be significantly improved by taking account of codon substitutions with multiple nucleotide changes [37], [38].

### A Method Based on Co-substitutions between Sites Rather than the Joint Distributions of Residue Types

So far remarkable improvements in the accuracy of contact prediction were all achieved by extracting essential correlations of amino acid types between residue positions from multiple sequence alignments [11], [15], [16], [32]. Here, almost comparable accuracy of contact prediction has been achieved by evaluating direct correlations of concurrent and compensatory substitutions between sites. The present method cannot be applied to the cases in which all substitutions are nearly neutral. In such a case, in which pairwise interactions between sites are not significant and multi-body interactions among sites are important to stabilize a conformation, structural and functional constraints from closely-located sites in protein 3D structures may be reflected only in the joint distribution of amino acid types between the sites.

Residue-residue interactions maintaining secondary structures appear to be more easily detected by the joint distribution of amino acid types between the sites than concurrent substitutions. In general, the present method less detects secondary structure interactions between neighboring sites along a sequence than the other. Marks et al. [16] reported that residue pairs separated by four or five positions in a sequence often have high DI scores without being in close physical proximity in the folded protein. Even for site pairs separated by more than five positions, their method based on the joint distribution of amino acid types detected more dependences in 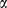 helical regions than the present method; see 7tm_1 in Table 5 and in Fig. 4.

From a such viewpoint, methods of extracting direct correlations of amino acid types between sites may be better for extracting direct dependences between sites than those of detecting compensatory substitutions in a tree. However, interactions between closely-located sites do not necessarily result in distinct correlations of amino acid types between the sites. Residue-residue interactions that are less specific to amino acid type are such interactions. For example, hydrophobic interactions are relatively non-specific, but significantly contribute to residue-residue interactions inside protein structures. In the case of membrane proteins, most of amino acids embedded in membrane are hydrophobic. Even in the case that residue-residue interactions are too non-specific to result in distinct correlations of amino acid types between sites, physico-chemical changes due to substitutions may require compensatory substitutions, and therefore the interactions may be identified by detecting compensatory substitutions. Membrane proteins may be this case; see 7tm_1 in Table 5 and in Fig. 3. Structural analyses of membrane proteins, especially the determinations of protein coordinates in transmembrane regions, are difficult in comparison of globular proteins. The present method for contact prediction could facilitate the structure determination of membrane proteins.

The DI method based on the joint distributions of amino acid types may be simpler and faster than the present method based on co-substitutions in a phylogenetic tree. However, the joint distributions of amino acid types calculated from a multiple sequence alignment include more or less phylogenetic bias, but there is no such a bias in the present method. Thus, the both types of methods are complementary to each other.

### A Method Based on a Gaussian Graphical Model Rather than a Bayesian Graphical Model

A Bayesian graphical model was applied to disentangling direct from indirect dependencies between residue positions in multiple sequence alignments of proteins [11]. In the Bayesian graphical model, an acyclic directed graph is assumed for site dependence, although interactions between sites in protein structures act on each other. A causal relationship between substitutions is of course directional. However, substitutions at a site affect on closely-located sites, and also the site is affected by substitutions at those surrounding sites. Thus, dependence between sites should be assumed to be bidirectional or undirectional. Unlike Bayesian graphical models, an undirected graph is assumed in a Gaussian graphical model [40], in which a null edge between two nodes encodes that random variables assigned to the nodes are conditionally independent of each other given the values of random variables assigned to other nodes. Assuming that a joint probability density distribution of random variables is a multivariate Gaussian distribution, two random variables are conditionally independent given the values of other random variables if and only if a partial correlation coefficient between the two random variables is equal to zero. Thus, the present model based on partial correlation coefficients can be regarded as a Gaussian graphical model in which an undirected graph is assumed for dependences between sites and a feature vector 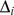 is assigned to node 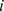 as the observed values of a random variable. This is one of essential differences between the present model and the Bayesian models [11], [14], [29], although there is another essential difference that the joint distributions of residues at sites were analyzed in [11], [14].

### Contribution to Protein Structure Prediction

Determination of protein structure is essential to understand protein function. However, despite significant effort to explore unknown folds in the protein structural space, protein structures determined by experiment are far less than known protein families. Only about 36% of the Pfam manually curated families (Pfam-A, 13672 families) include at least one member whose structure is known. In the case of domains of unknown function (DUFs), which are rapidly growing in the Pfam-A, some 26% of DUFs have at least one structurally determined protein within a family or within a clan [35]. On the other hand, the Pfam automatically generated database (Pfam-B), which may be regarded as an upper limit for the total number of protein families, amounts to 460125 families. The number and also the size of protein families will further grow as genome/metagenome sequencing projects proceed with next-generation sequencing technologies. Thus, accurate *de novo* prediction of three-dimensional structure is desirable to catch up with the high growing speed of protein families with unknown folds.

The vast conformational space of protein makes it difficult to determine protein structure by *ab initio* folding of protein. Methods that use fragment libraries [64], [65] or other strategies with statistical potentials [66] to efficiently search conformational space have been quite successful in *de novo* prediction of protein structure, but their conformational samplings are not efficient enough to fold longer proteins than at most 100 residues.

On the other hand, the accuracy of the present contact prediction is insensitive to sequence length; see Table 5. Also, the increase of protein family size is beneficial to the contact prediction from evolutionary sequence variation. Thus, contact residue pairs predicted from a statistical analysis of a multiple sequence alignment and/or from concurrent and compensatory substitutions are useful as distance constraints in structure prediction [67]. It is shown [16], [32] that inferred residue-residue proximities together with a predicted secondary structure provide sufficient information to predict a protein fold without the use of known three-dimensional structures.

The present contact prediction based on coevolving site pairs is comparable to the method [16] based on the joint distribution of amino acids in a multiple sequence alignment, but better for a membrane protein (7tm_1) although the prediction accuracy is not high. Thus, the present method is especially useful for the determination of the arrangement of trans-membrane segments in membrane proteins whose structure determination by experiment is relatively difficult.

## Supporting Information

Figure S1
**Dependence of PPV on the number of characteristic variables used.** For each protein in 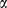, 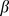, 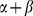, and 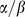 folds, PPVs are plotted against the number of characteristic variables used to score co-substitutions between sites. The characteristic variables except 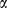 propensity listed in Table 4 are added in the listed order to define an overall coevolution score; that is, (1) occurrence of amino acid substitution, (2) side-chain volume, (3) charge, (4) hydrogen-bonding capability, (5) hydrophobicity, (6) 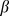 and (7) turn propensities, (8) aromatic interaction, (9) branched side-chain, (10) cross-link capability, and (11) ionic side-chain. The solid and dotted lines correspond to predictions in which the ratio of the predicted to the true contacts is equal to 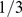 or 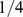, respectively.(PDF)

Figure S2
**Coevolving site pairs versus DI residue pairs.** Residue pairs whose minimum atomic distances are shorter than 5 Å in a protein structure and coevolving site pairs predicted are shown by gray filled-squares and by red or indigo filled-circles in the lower-left half of each figure, respectively. For comparison, such residue-residue proximities and predicted contact residue pairs with high DI scores in [16] are shown by gray filled-squares and by red or indigo filled-circles in the upper-right half of each figure, respectively; only the conservation filter is applied but the filters based on a secondary structure prediction and for cysteine pairs are not applied to the DI scores. Red and indigo filled-circles correspond to true and false contact residue pairs, respectively. Residue pairs separated by five or fewer positions (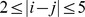) in a sequence may be shown with the gray filled-squares but are excluded as well as nearest neighbors in both the predictions. The total numbers of coevolving site pairs and DI residue pairs plotted for each protein are both equal to one third of true contacts (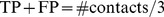). The PPVs of both the methods for each protein are listed in Table 5.(PDF)

Figure S3
**Dependence of MDPNT on the number of predicted contacts.** The dependences of the mean Euclidean distance from predicted site pairs to the nearest true contact in the 2-dimensional sequence-position space on the total number of predicted contacts are shown for each protein fold of 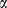, 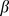, 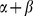, and 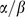. The solid and dotted lines show the MDPNTs of the present method and the method based on the DI score [16], respectively. Only the conservation filter [16] is applied for the DI score. The total number of predicted contacts is shown in the scale of the ratio of the number of predicted contacts to the number of true contacts. The total number of predicted site pairs takes every 10 from 10 to a sequence length; also MDPNTs for the numbers of predicted contacts equal to one fourth or one third of true contacts are plotted. The filled marks indicate the points corresponding to the number of predicted site pairs equal to one third of the number of true contacts. The number of sequences used here for each protein family is one listed in Table 1.(PDF)

Figure S4
**Dependence of MDTNP on the number of predicted contacts.** The dependences of the mean Euclidean distance from every true contact to the nearest predicted site pair in the 2-dimensional sequence-position space on the total number of predicted contacts are shown for each protein fold of 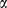, 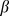, 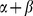, and 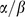. The solid and dotted lines show the MDTNPs of the present method and the method based on the DI score [16], respectively. Only the conservation filter [16] is applied for the DI score. The total number of predicted site pairs is shown in the scale of the ratio of the number of predicted site pairs to the number of true contacts. The total number of predicted site pairs takes every 10 from 10 to a sequence length; also MDTNPs for the numbers of predicted site pairs equal to one fourth or one third of true contacts are plotted. The filled marks indicate the points corresponding to the number of predicted contacts equal to one third of the number of true contacts. The number of sequences used here for each protein family is one listed in Table 1.(PDF)

Figure S5
**Dependence of MDPNT on the number of sequences used.** The mean Euclidean distance from every predicted site pair to the nearest true contact in the 2-dimensional sequence-position space is plotted against the total number of homologous sequences used for each prediction. The total numbers of coevolving site pairs predicted for each protein are equal to one third of true contacts. The filled marks indicate the points corresponding to the number of used sequences listed for each protein family in Table 1. The values written near each data point indicate the threshold value 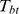; OTUs connected to their parent nodes with branches shorter than this threshold value are removed in the Pfam reference tree of the Pfam full sequences used for each prediction. Some data points correspond to datasets generated by using the same value of the threshold but by removing different OTUs.(PDF)

Figure S6
**Dependence of MDTNP on the number of sequences used.** The mean Euclidean distance from every true contact to the nearest predicted site pair in the 2-dimensional sequence-position space is plotted against the total number of homologous sequences used for each prediction. The total numbers of coevolving site pairs predicted for each protein are equal to one third of true contacts. The filled marks indicate the points corresponding to the number of used sequences listed for each protein family in Table 1. The values written near each data point indicate the threshold value 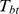; OTUs connected to their parent nodes with branches shorter than this threshold value are removed in the Pfam reference tree of the Pfam full sequences used for each prediction. Some data points correspond to datasets generated by using the same value of the threshold but by removing different OTUs.(PDF)

Table S1 Dependence of contact prediction accuracies on phylogenetic trees.
(PDF)

Data S1 **Coevolution scores, overall coevolution score and rank of each site pair in each protein.**
(BZ2)
