# Supplementary material for: Prediction of Contact Residue Pairs Based on Co-Substitution between Sites in Protein Structures
Source: PLoS One. 2013 Jan 16;8(1):e54252. doi: 10.1371/journal.pone.0054252 (PMC3546969; doi:10.1371/journal.pone.0054252)
Supplement: Figure S1 — Dependence of PPV on the number of characteristic variables used. For each protein in , , , and folds, PPVs are plotted against the number of characteristic variables used to score co-substitutions between sites. The characteristic variables except propensity listed in Table 4 are added in the listed order to define an overall coevolution score; that is, (1) occurrence of amino acid substitution, (2) side-chain volume, (3) charge, (4) hydrogen-bonding capability, (5) hydrophobicity, (6) and (7) turn propensities, (8) aromatic interaction, (9) branched side-chain, (10) cross-link capability, and (11) ionic side-chain. The solid and dotted lines correspond to predictions in which the ratio of the predicted to the true contacts is equal to or , respectively. (PDF) [file pone.0054252.s001.pdf]

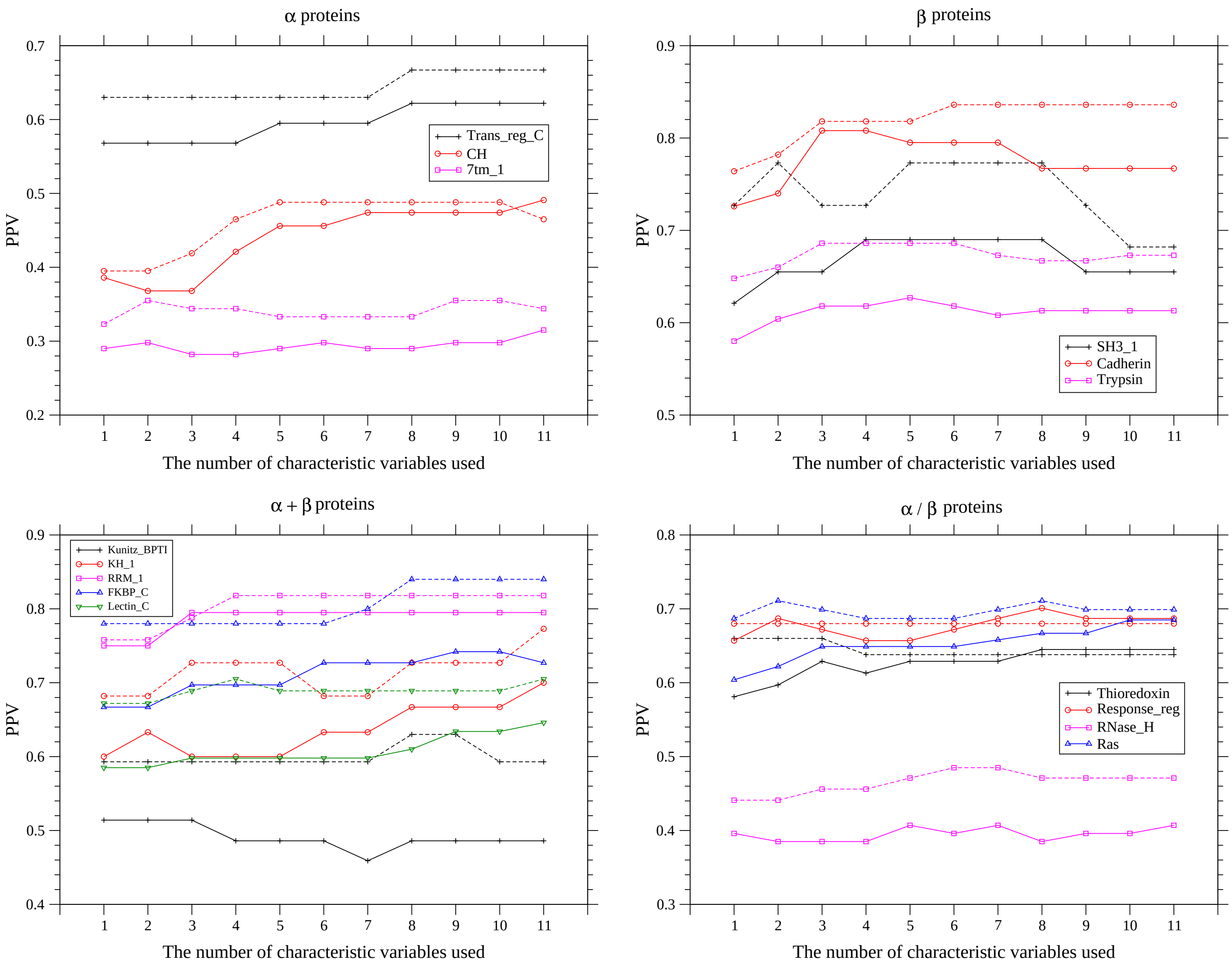

Figure S1. Dependence of PPV on the number of characteristic variables used. For each protein in α, β, $\alpha+\beta$, and α/β folds, PPVs are plotted against the number of characteristic variables used to score co-substitutions between sites. The characteristic variables except α propensity listed in Table 4 are added in the listed order to define an overall coevolution score; that is, (1) occurrence of amino acid substitution, (2) side-chain volume, (3) charge, (4) hydrogen-bonding capability, (5) hydrophobicity, (6) β and (7) turn propensities, (8) aromatic interaction, (9) branched side-chain, (10) cross-link capability, and (11) ionic side-chain. The solid and dotted lines correspond to predictions in which the ratio of the predicted to the true contacts is equal to $1/3$ or $1/4$, respectively.
