# Supplementary material for: Prediction of Contact Residue Pairs Based on Co-Substitution between Sites in Protein Structures
Source: PLoS One. 2013 Jan 16;8(1):e54252. doi: 10.1371/journal.pone.0054252 (PMC3546969; doi:10.1371/journal.pone.0054252)
Supplement: Figure S2 — Coevolving site pairs versus DI residue pairs. Residue pairs whose minimum atomic distances are shorter than 5 Å in a protein structure and coevolving site pairs predicted are shown by gray filled-squares and by red or indigo filled-circles in the lower-left half of each figure, respectively. For comparison, such residue-residue proximities and predicted contact residue pairs with high DI scores in [16] are shown by gray filled-squares and by red or indigo filled-circles in the upper-right half of each figure, respectively; only the conservation filter is applied but the filters based on a secondary structure prediction and for cysteine pairs are not applied to the DI scores. Red and indigo filled-circles correspond to true and false contact residue pairs, respectively. Residue pairs separated by five or fewer positions () in a sequence may be shown with the gray filled-squares but are excluded as well as nearest neighbors in both the predictions. The total numbers of coevolving site pairs and DI residue pairs plotted for each protein are both equal to one third of true contacts (). The PPVs of both the methods for each protein are listed in Table 5. (PDF) [file pone.0054252.s002.pdf]

**Figure S2. Coevolving site pairs versus DI residue pairs.** Residue pairs whose minimum atomic distances are shorter than 5 Å in a protein structure and coevolving site pairs predicted are shown by gray filled-squares and by red or indigo filled-circles in the lower-left half of each figure, respectively. For comparison, such residue-residue proximities and predicted contact residue pairs with high DI scores in [16] are shown by gray filled-squares and by red or indigo filled-circles in the upper-right half of each figure, respectively; only the conservation filter is applied but the filters based on a secondary structure prediction and for cysteine pairs are not applied to the DI scores. Red and indigo filled-circles correspond to true and false contact residue pairs, respectively. Residue pairs separated by five or fewer positions ($2 \leq |i - j| \leq 5$) in a sequence may be shown with the gray filled-squares but are excluded as well as nearest neighbors in both the predictions. The total numbers of coevolving site pairs and DI residue pairs plotted for each protein are both equal to one third of true contacts ($\mathrm{TP} + \mathrm{FP} = \#\mathrm{contacts}/3$). The PPVs of both the methods for each protein are listed in Table 5.

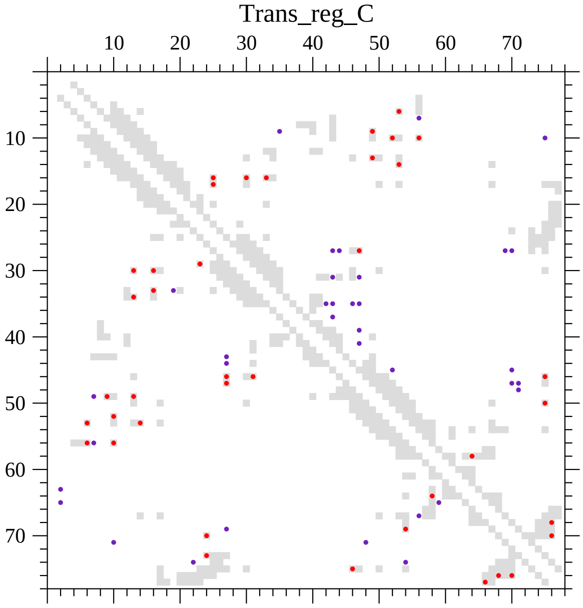
Trans_reg_C
10
20
30
40
50
60
70

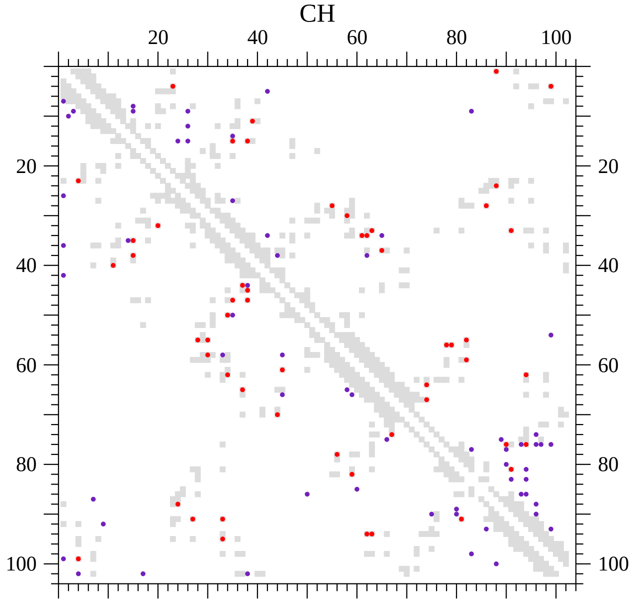
CH
20
40
60
80
100

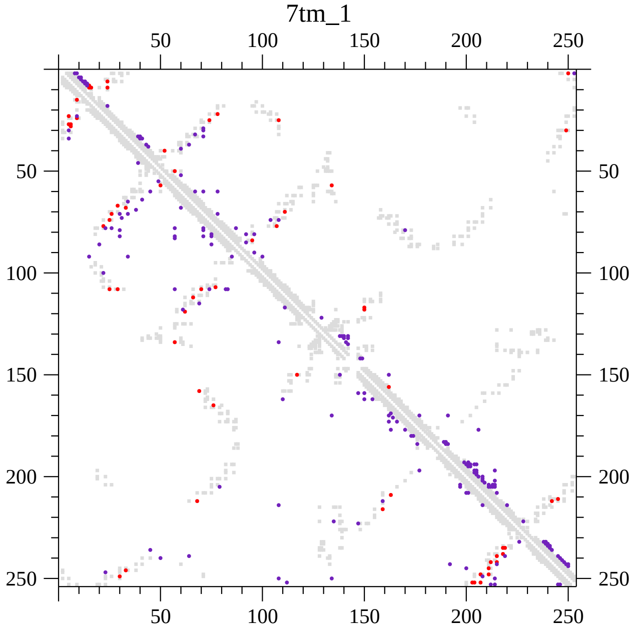
7tm_1
50
100
150
200
250

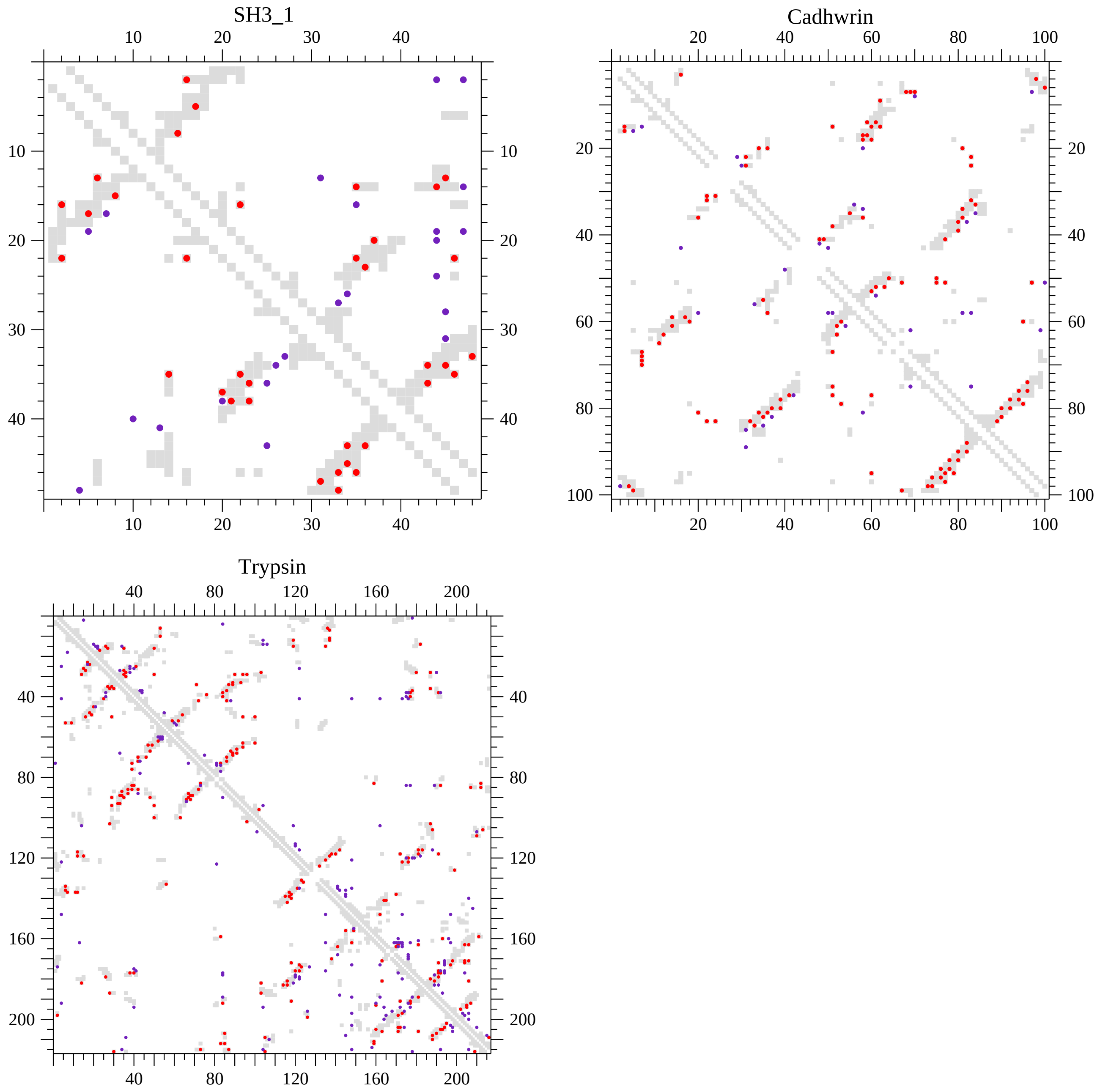
SH3_1
10
20
30
40
Cadhwrin
20
40
60
80
100
Trypsin
40
80
120
160
200

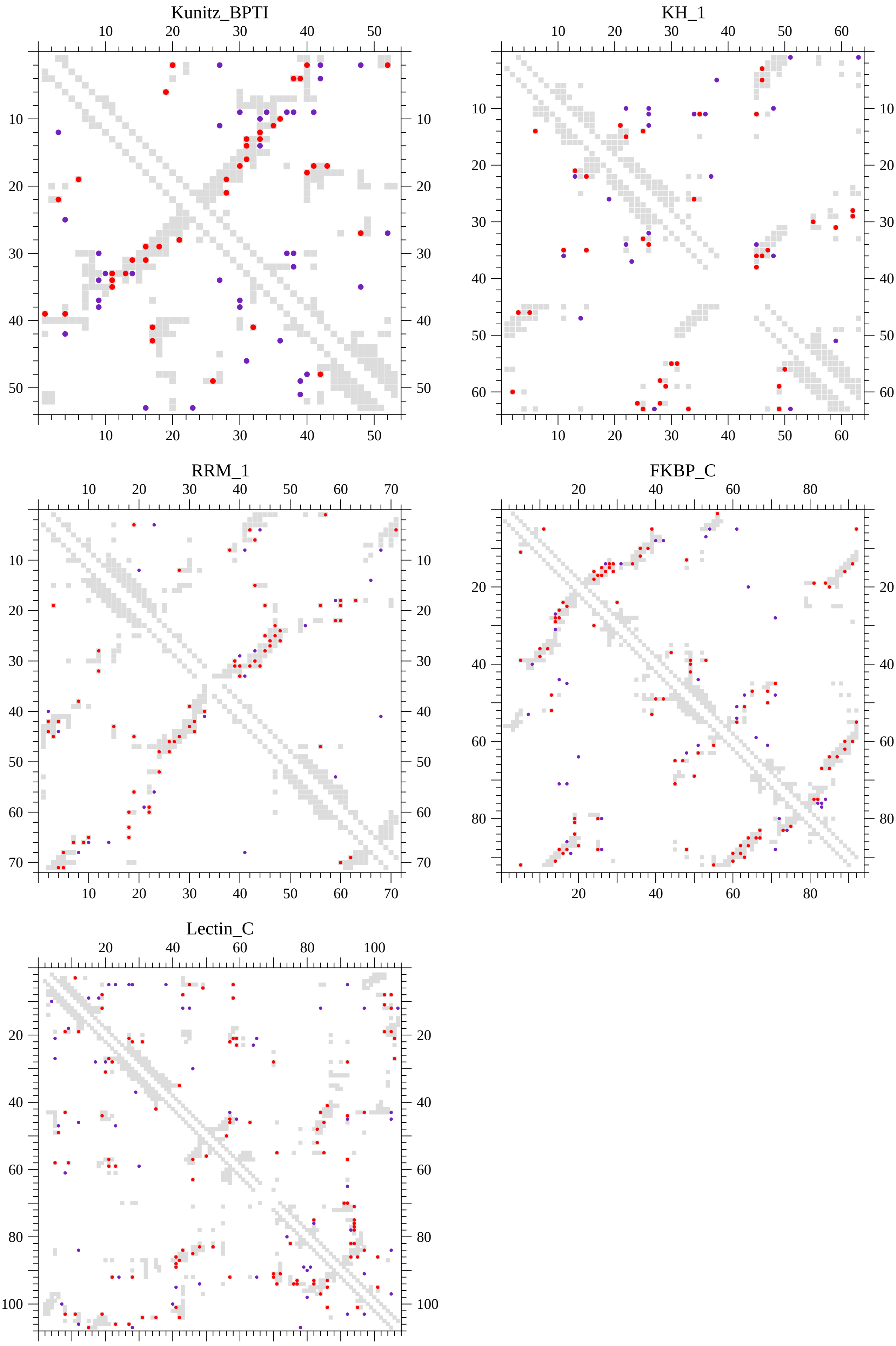
Kunitz_BPTI
KH_1
RRM_1
FKBP_C
Lectin_C

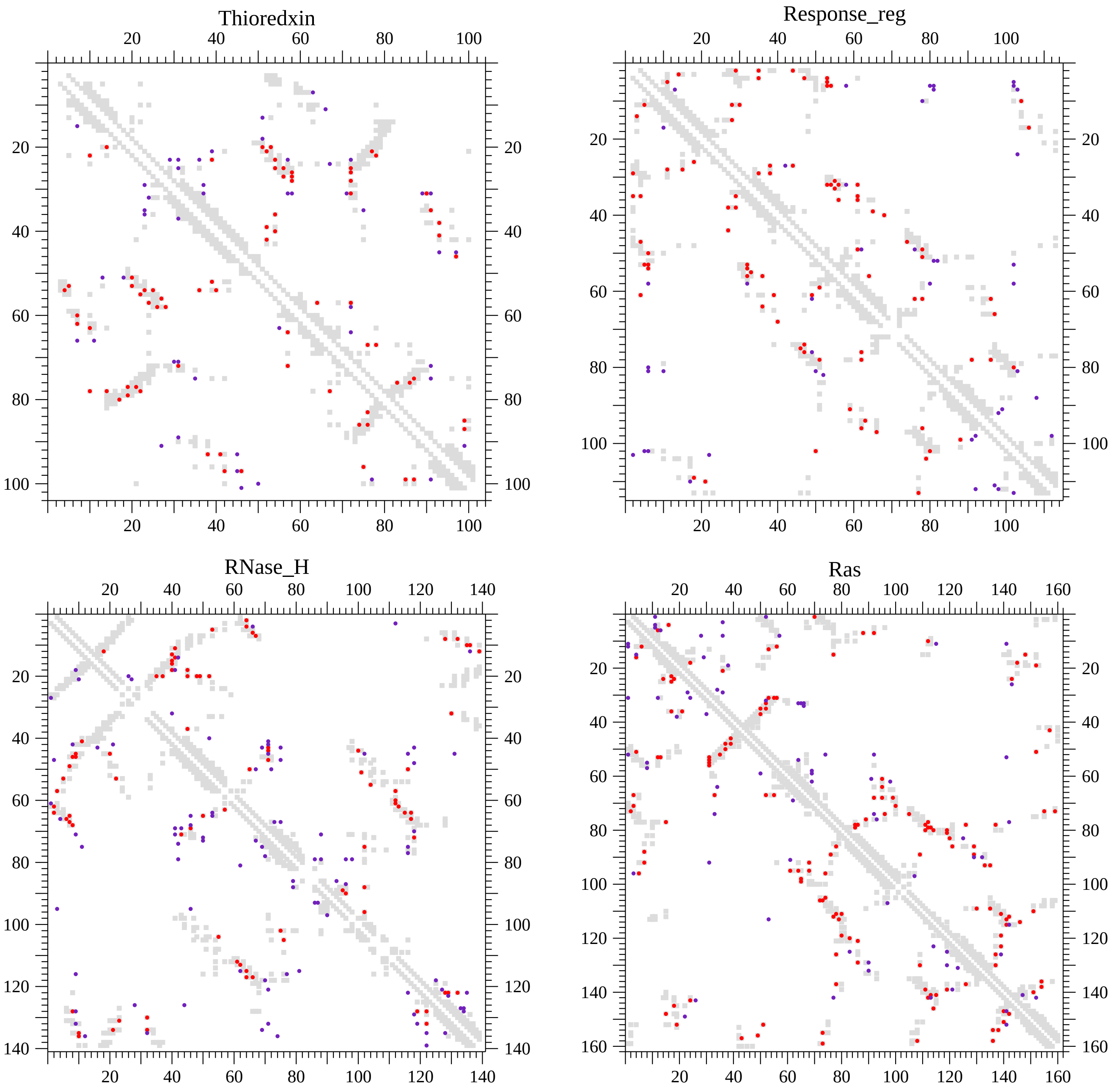

Thioredxin
Response_reg
RNase_H
Ras
