# Supplementary material for: Prediction of Contact Residue Pairs Based on Co-Substitution between Sites in Protein Structures
Source: PLoS One. 2013 Jan 16;8(1):e54252. doi: 10.1371/journal.pone.0054252 (PMC3546969; doi:10.1371/journal.pone.0054252)
Supplement: Figure S3 — Dependence of MDPNT on the number of predicted contacts. The dependences of the mean Euclidean distance from predicted site pairs to the nearest true contact in the 2-dimensional sequence-position space on the total number of predicted contacts are shown for each protein fold of , , , and . The solid and dotted lines show the MDPNTs of the present method and the method based on the DI score [16], respectively. Only the conservation filter [16] is applied for the DI score. The total number of predicted contacts is shown in the scale of the ratio of the number of predicted contacts to the number of true contacts. The total number of predicted site pairs takes every 10 from 10 to a sequence length; also MDPNTs for the numbers of predicted contacts equal to one fourth or one third of true contacts are plotted. The filled marks indicate the points corresponding to the number of predicted site pairs equal to one third of the number of true contacts. The number of sequences used here for each protein family is one listed in Table 1. (PDF) [file pone.0054252.s003.pdf]

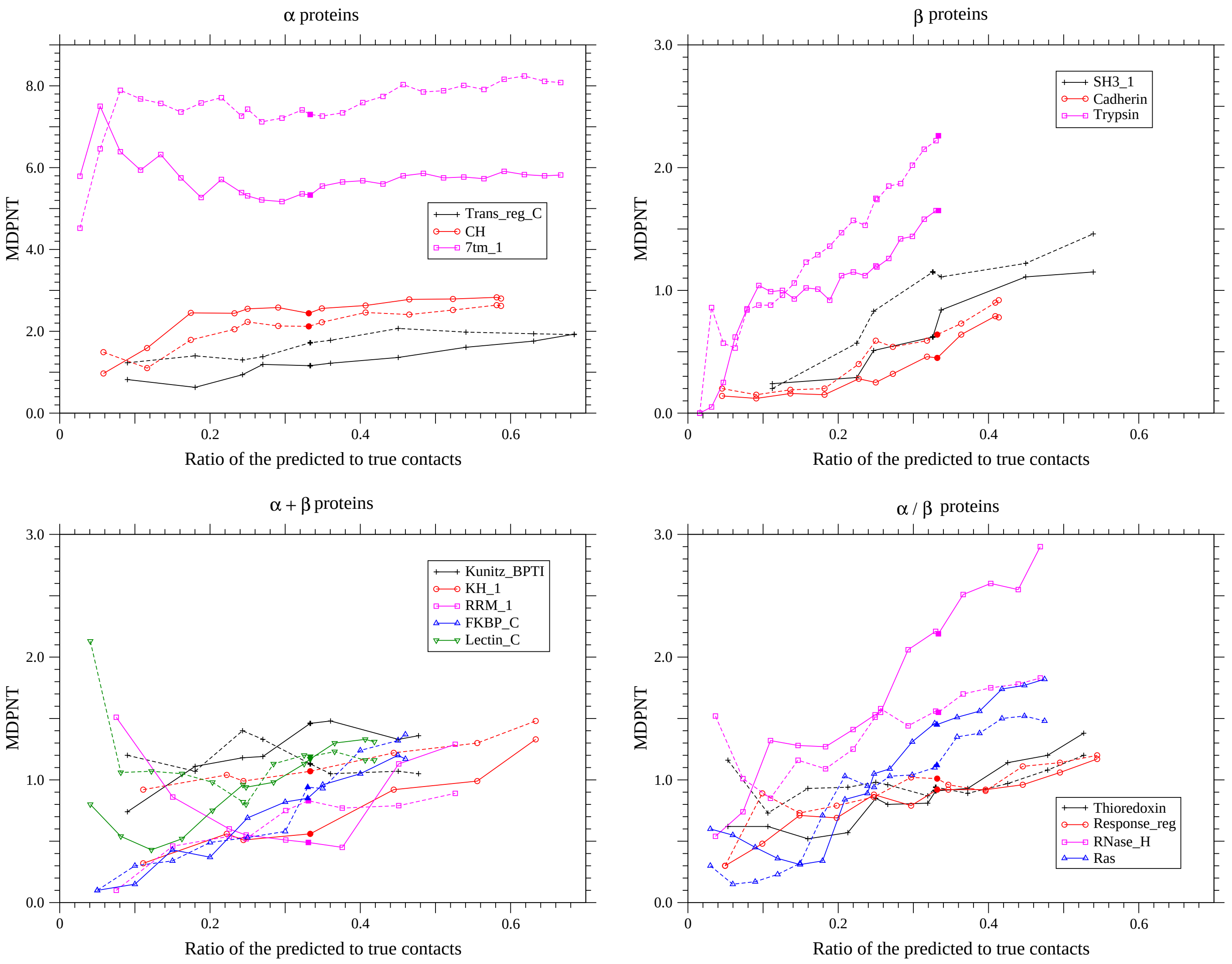

Figure S3. Dependence of MDPNT on the number of predicted contacts. The dependences of the mean Euclidean distance from predicted site pairs to the nearest true contact in the 2-dimensional sequence-position space on the total number of predicted contacts are shown for each protein fold of α, β, $\alpha+\beta$, and α/β. The solid and dotted lines show the MDPNTs of the present method and the method based on the DI score [16], respectively. Only the conservation filter [16] is applied for the DI score. The total number of predicted contacts is shown in the scale of the ratio of the number of predicted contacts to the number of true contacts. The total number of predicted site pairs takes every 10 from 10 to a sequence length; also MDPNTs for the numbers of predicted contacts equal to one fourth or one third of true contacts are plotted. The filled marks indicate the points corresponding to the number of predicted site pairs equal to one third of the number of true contacts. The number of sequences used here for each protein family is one listed in Table 1.
