# Supplementary material for: Prediction of Contact Residue Pairs Based on Co-Substitution between Sites in Protein Structures
Source: PLoS One. 2013 Jan 16;8(1):e54252. doi: 10.1371/journal.pone.0054252 (PMC3546969; doi:10.1371/journal.pone.0054252)
Supplement: Figure S4 — Dependence of MDTNP on the number of predicted contacts. The dependences of the mean Euclidean distance from every true contact to the nearest predicted site pair in the 2-dimensional sequence-position space on the total number of predicted contacts are shown for each protein fold of , , , and . The solid and dotted lines show the MDTNPs of the present method and the method based on the DI score [16], respectively. Only the conservation filter [16] is applied for the DI score. The total number of predicted site pairs is shown in the scale of the ratio of the number of predicted site pairs to the number of true contacts. The total number of predicted site pairs takes every 10 from 10 to a sequence length; also MDTNPs for the numbers of predicted site pairs equal to one fourth or one third of true contacts are plotted. The filled marks indicate the points corresponding to the number of predicted contacts equal to one third of the number of true contacts. The number of sequences used here for each protein family is one listed in Table 1. (PDF) [file pone.0054252.s004.pdf]

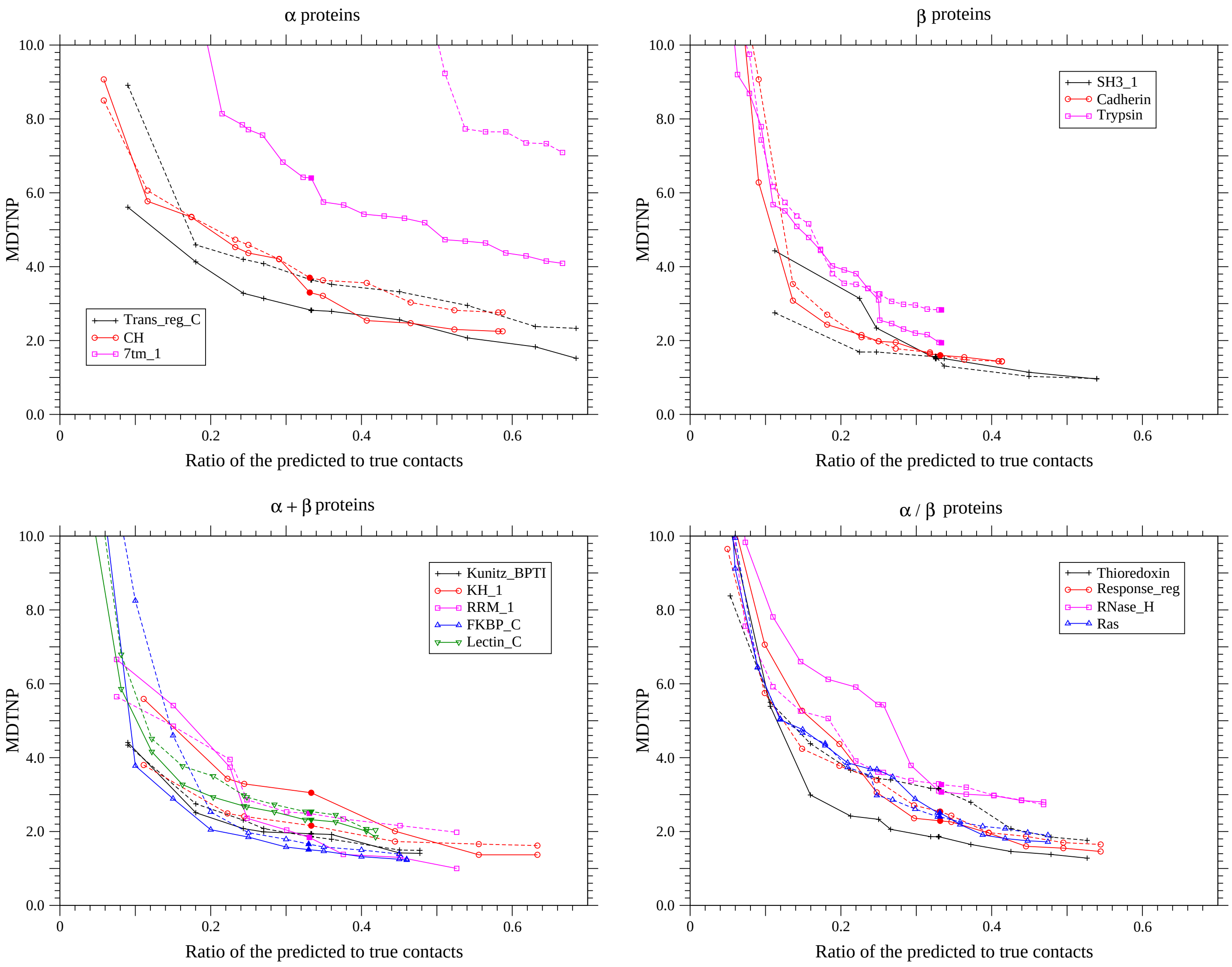

Figure S4. Dependence of MDTNP on the number of predicted contacts. The dependences of the mean Euclidean distance from every true contact to the nearest predicted site pair in the 2-dimensional sequence-position space on the total number of predicted contacts are shown for each protein fold of α, β, $\alpha + \beta$, and α/β. The solid and dotted lines show the MDTNPs of the present method and the method based on the DI score [16], respectively. Only the conservation filter [16] is applied for the DI score. The total number of predicted site pairs is shown in the scale of the ratio of the number of predicted site pairs to the number of true contacts. The total number of predicted site pairs takes every 10 from 10 to a sequence length; also MDTNPs for the numbers of predicted site pairs equal to one fourth or one third of true contacts are plotted. The filled marks indicate the points corresponding to the number of predicted contacts equal to one third of the number of true contacts. The number of sequences used here for each protein family is one listed in Table 1.
