# Supplementary material for: Prediction of Contact Residue Pairs Based on Co-Substitution between Sites in Protein Structures
Source: PLoS One. 2013 Jan 16;8(1):e54252. doi: 10.1371/journal.pone.0054252 (PMC3546969; doi:10.1371/journal.pone.0054252)
Supplement: Figure S5 — Dependence of MDPNT on the number of sequences used. The mean Euclidean distance from every predicted site pair to the nearest true contact in the 2-dimensional sequence-position space is plotted against the total number of homologous sequences used for each prediction. The total numbers of coevolving site pairs predicted for each protein are equal to one third of true contacts. The filled marks indicate the points corresponding to the number of used sequences listed for each protein family in Table 1. The values written near each data point indicate the threshold value ; OTUs connected to their parent nodes with branches shorter than this threshold value are removed in the Pfam reference tree of the Pfam full sequences used for each prediction. Some data points correspond to datasets generated by using the same value of the threshold but by removing different OTUs. (PDF) [file pone.0054252.s005.pdf]

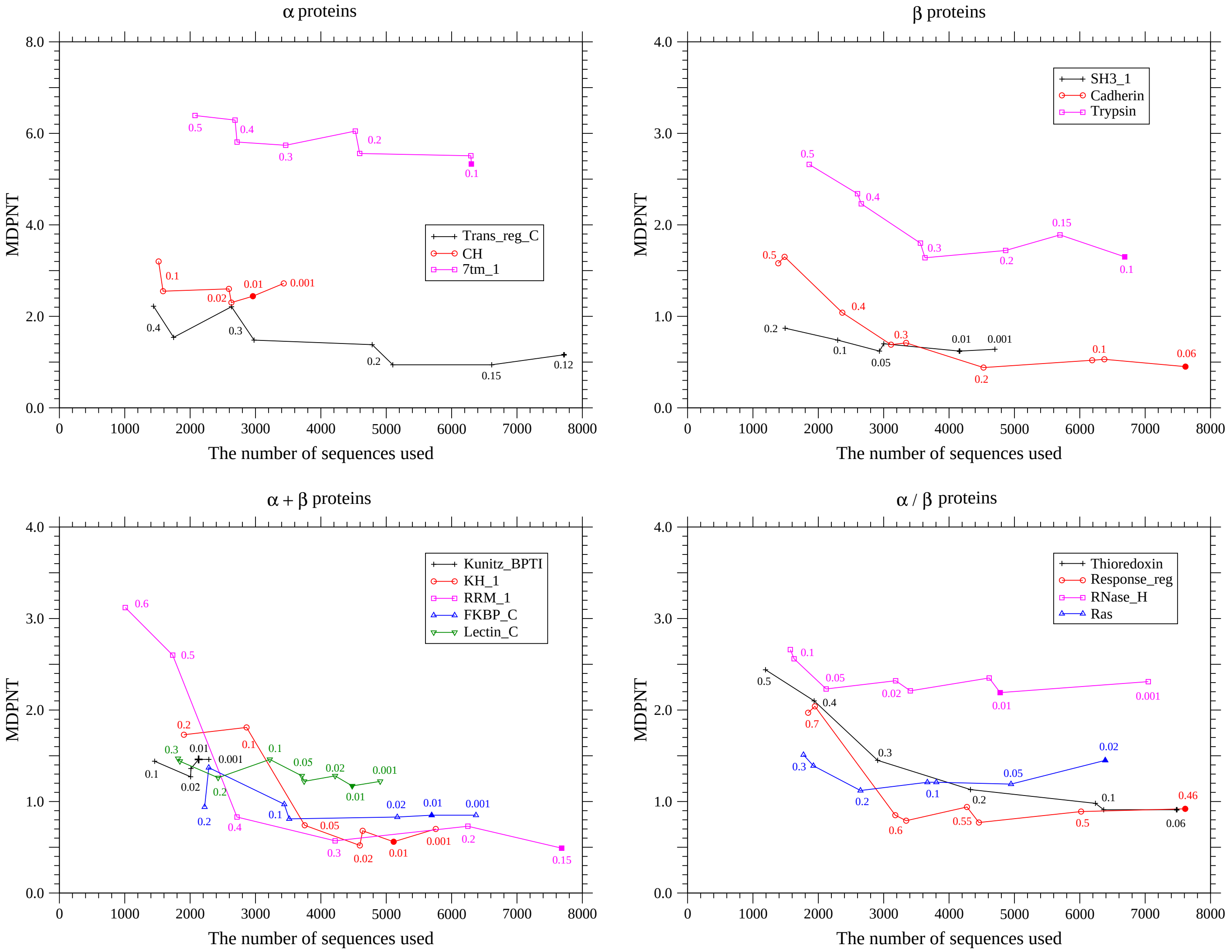

Figure S5. Dependence of MDPNT on the number of sequences used. The mean Euclidean distance from every predicted site pair to the nearest true contact in the 2-dimensional sequence-position space is plotted against the total number of homologous sequences used for each prediction. The total numbers of coevolving site pairs predicted for each protein are equal to one third of true contacts. The filled marks indicate the points corresponding to the number of used sequences listed for each protein family in Table 1. The values written near each data point indicate the threshold value T_{bt}; OTUs connected to their parent nodes with branches shorter than this threshold value are removed in the Pfam reference tree of the Pfam full sequences used for each prediction. Some data points correspond to datasets generated by using the same value of the threshold but by removing different OTUs.
