# Supplementary material for: Prediction of Contact Residue Pairs Based on Co-Substitution between Sites in Protein Structures
Source: PLoS One. 2013 Jan 16;8(1):e54252. doi: 10.1371/journal.pone.0054252 (PMC3546969; doi:10.1371/journal.pone.0054252)
Supplement: Table S1 — Dependence of contact prediction accuracies on phylogenetic trees. (PDF) [file pone.0054252.s007.pdf]

Table S1. Dependence of contact prediction accuracies on phylogenetic trees.

Pfam ID*	#contacts /#sites**	TP + FP***	DI §§	PPV§ $\rho_{ij}^{\S\S\S}$ Pfam tree †	FastTree2 ††	ExaML ††	Relative log-likelihood‡ Pfam tree †	FastTree2 ††	ExaML †††
Trans_reg_C	111/76	27	0.556	**0.667**	**0.667**		(−772541.8)	2768.9	
	1.5	37	0.432	**0.622**	0.595				
CH	172/101	43	**0.488**	0.465	0.419	0.395	(−246974.5)	1818.6	2988.1
	1.7	57	0.439	**0.491**	0.456	0.351			
7tm_1	372/248	93	0.194	0.344	**0.366**		(−1971205.1)	44545.9	
	1.5	124	0.169	**0.306**	**0.306**				
SH3_1	89/48	22	0.636	**0.682**	**0.682**	**0.682**	(−178181.5)	1214.8	2566.5
	1.9	29	0.552	0.655	0.586	**0.690**			
Cadherin	220/91	55	0.818	**0.836**	0.800		(−917754.4)	2891.1	
	2.4	73	0.753	**0.767**	0.740				
Trypsin	636/212	159	0.591	**0.673**	0.648		(−1843495.9)	5728.3	
	3.0	212	0.533	**0.613**	0.604				
Kunitz_BPTI	111/53	27	0.444	**0.593**	0.556	0.556	(−127989.5)	600.6	1731.1
	2.1	37	**0.541**	0.486	0.514	0.514			
KH_1	90/57	22	0.500	0.773	**0.818**		(−253902.4)	2428.0	
	1.6	30	0.533	**0.700**	**0.700**				
RRM_1	133/70	33	0.758	**0.818**	0.788		(−780196.4)	3056.8	
	1.9	44	0.705	**0.795**	0.773				
FKBP_C	200/92	50	0.760	**0.840**	0.800		(−455605.4)	3935.5	
	2.2	66	0.697	0.727	**0.773**				
Lectin_C	246/103	61	**0.770**	0.705	0.705		(−555599.9)	3073.6	
	2.4	82	**0.671**	0.646	0.610				
Thioredoxin	188/99	47	0.532	0.638	**0.660**		(−926791.5)	4137.4	
	1.9	62	0.565	**0.645**	**0.645**				
Response_reg	202/110	50	0.660	0.680	**0.700**		(−1654255.6)	2934.4	
	1.8	67	0.642	0.687	**0.716**				
RNase_H	273/128	68	**0.559**	0.471	0.456		(−364080.9)	4787.3	
	2.1	91	**0.549**	0.407	0.407				
Ras	335/159	83	0.699	0.699	**0.723**		(−932720.7)	9667.8	
	2.1	111	0.631	**0.685**	0.667				

* The threshold T_{bt} to remove OTUs with short branches and the number n_{otu} of remaining OTUs that are used for each protein here are listed in Table 2.

** The numbers of contacts and of sites, and their ratio are listed. Protein structures used to calculate contact residue pairs are listed in Table 1. Neighboring residue pairs within 5 residues ($|i-j| \leq 5$) along a peptide chain are excluded in the evaluation of prediction accuracy.

*** TP and FP are the numbers of true and false positives, and their sum is equal to the number of predicted contacts; only predictions for TP + FP = #contacts/4 and #contacts/3 are listed.

§ PPV stands for a positive predictive value; i.e., PPV = TP/(TP + FP). Better values are typed in a bold font.

§§ DI means the prediction based on the direct information (DI) score published in [16]; a filtering based on a secondary structure prediction is not applied but only a conservation filter [16] is.

§§§ Predictions based on the coevolution score.

† Pfam reference philogenetic trees are used.

†† Philogenetic trees optimized by FastTree2 [61] with the default option (JTT and CAT) for datasets of $T_{bt} = 0.01$ or full alignments are used as tree topologies for these protein alignments whose T_{bt} values are listed in Table 2.

††† Philogenetic trees optimized by FastTree2 [61] and then ExaML [62] with the default option (JTT and CAT) for datasets of $T_{bt} = 0.01$ or full alignments are used as tree topologies for these protein alignments whose T_{bt} values are listed in Table 2.

‡ The log-likelihood of a tree with branch lengths optimized in a codon model for each topology of the Pfam reference tree, an appropriate ML tree by FastTree2 [61], and a maximum-likelihood tree by ExaML [62] is listed in relative to the log-likelihood of the Pfam reference tree whose value is written in parenthesis.
